# High-quality semiconductor fibres via mechanical design

**DOI:** 10.1038/s41586-023-06946-0

**Published:** 2024-01-31

**Authors:** Zhixun Wang, Zhe Wang, Dong Li, Chunlei Yang, Qichong Zhang, Ming Chen, Huajian Gao, Lei Wei

**Affiliations:** 1 School of Electrical and Electronic Engineering, Nanyang Technological University, Singapore, Singapore; 2 Key Laboratory of Bionic Engineering (Ministry of Education), Jilin University, Changchun, China; 3 School of Mechanical and Aerospace Engineering, Nanyang Technological University, Singapore, Singapore; 4 University of Chinese Academy of Sciences, Beijing, China; 5 Shenzhen Institute of Advanced Technology, Chinese Academy of Sciences, Shenzhen, China; 6 Key Laboratory of Multifunctional Nanomaterials and Smart Systems, Suzhou Institute of Nano-Tech and Nano-Bionics, Chinese Academy of Sciences, Suzhou, China; 7 Institute of High-Performance Computing, Agency for Science, Technology and Research, Singapore, Singapore; 8 Institute for Digital Molecular Analytics and Science (IDMxS), Nanyang Technological University, Singapore, Singapore

**Keywords:** Electronic devices, Mechanical engineering

## Abstract

Recent breakthroughs in fibre technology have enabled the assembly of functional materials with intimate interfaces into a single fibre with specific geometries^1–11^, delivering diverse functionalities over a large area, for example, serving as sensors, actuators, energy harvesting and storage, display, and healthcare apparatus^12–17^. As semiconductors are the critical component that governs device performance, the selection, control and engineering of semiconductors inside fibres are the key pathways to enabling high-performance functional fibres. However, owing to stress development and capillary instability in the high-yield fibre thermal drawing, both cracks and deformations in the semiconductor cores considerably affect the performance of these fibres. Here we report a mechanical design to achieve ultralong, fracture-free and perturbation-free semiconductor fibres, guided by a study on stress development and capillary instability at three stages of the fibre formation: the viscous flow, the core crystallization and the subsequent cooling stage. Then, the exposed semiconductor wires can be integrated into a single flexible fibre with well-defined interfaces with metal electrodes, thereby achieving optoelectronic fibres and large-scale optoelectronic fabrics. This work provides fundamental insights into extreme mechanics and fluid dynamics with geometries that are inaccessible in traditional platforms, essentially addressing the increasing demand for flexible and wearable optoelectronics.

## Abstract (web summary)

A mechanical design is developed for the fabrication of ultralong, fracture-free and perturbation-free semiconductor fibres to address the increasing demand for flexible and wearable optoelectronics.

## Main

**Fig. 1:**
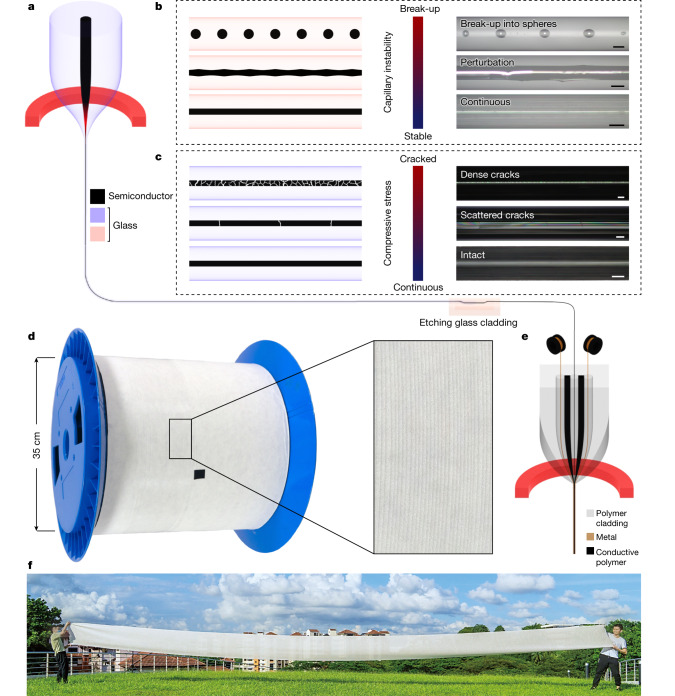
Design and fabrication of semiconductor optoelectronic fibres. **a**, Schematic of the molten-core method. The preform, made of glass cladding (purple) and semiconductor core (black), is drawn down to the fibre dimension. **b**, Fibre core geometries correspond to the development of capillary instability. Schematics (left) and optical images (right) of fibres with different degrees of capillary instability are shown. Scale bars, 100 μm. **c**, Fibre core geometries relate to different stress levels. Schematics (left) and optical images (right) of fibres show the intact and cracked cores in response to the stress formed during fabrication. Scale bars, 100 μm. **d**, About one-hundred metres of continuous semiconductor core fibre fabricated from one drawing process. The glass cladding can be removed by acid etching. **e**, Schematic of the convergence fibre drawing technique. Exposed semiconductor fibres and metal wires maintain their solidity during the process. Intimate interfaces of materials are formed in the necking region. **f**, Large-scale functional fabric enabled by the resulting optoelectronic fibres.

Glassy semiconductors are commonly used in thermally drawn fibres owing to their low processing temperatures and controllable fluidic behaviours^18–20^. However, compared with crystalline semiconductors that are most widely used in electronics, such as silicon (Si) and germanium (Ge), their inevitable high densities of electronic defects always lead to poor electrical properties of the fabricated fibres. Thus, the use of crystalline semiconductors is more favourable to fundamentally boost the further development of functional fibres. To obtain continuous long crystalline semiconductor fibres, various crystal growth techniques have been developed, such as the Czochralski, Bridgman–Stockbarger, float zone and micro-pulling-down methods^21–23^. Nonetheless, the growth rates and fabrication lengths are commonly limited to a few centimetres per hour and tens of centimetres. To achieve high-yield production of semiconductor fibres at extended lengths, the molten-core method was demonstrated^24^. Using this method, the semiconductor core is melted into a fluid flow that is confined by the glass cladding and thermally drawn into fibres (Fig. 1a). Over hundreds of metres of semiconductor fibre can thus be produced at the speed of a few tens of metres per minute in a single drawing process. The interface between the glass cladding and the semiconductor core contributes substantially to the complex stress development in the core, leading to either perturbed or fractured fibres (Fig. 1b,c), which primarily restricts the large-scale production of functional fibres. Despite the continuous efforts to optimize this method^25–28^, a thorough mechanical study on each stage of the fibre formation is lacking for establishing rational mechanical design to achieve ultralong, continuous, perturbation-free and fracture-free semiconductor fibres^29^.

Here we establish a theoretical system to clearly understand the morphology and stress development at the three stages of the fibre formation by the molten-core method: the viscous flow, the core crystallization and the subsequent cooling stage. We then proactively employ this in a mechanical design to achieve continuous high-quality semiconductor fibres (Fig. 1d). We further demonstrate optoelectronic fibres made of Si and Ge with single-core and dual-core structures via the convergence fibre drawing (Fig. 1e). Taking advantage of its form factor, the optoelectronic fibres cover a long sensing length and conform to curved surfaces where traditional rigid and discrete photo-detecting devices cannot be applied. The mechanical robustness of the optoelectronic fibres allows them to be woven into large-scale functional fabrics while maintaining favourable features such as conformability, washability and permeability (Fig. 1f). Such optoelectronic fibres offer a comparable performance to commercial planar-type photodetectors and enable diverse applications, for example, in healthcare, robotics, wearable communications and assistive technology.

## Stress formation

**Fig. 2:**
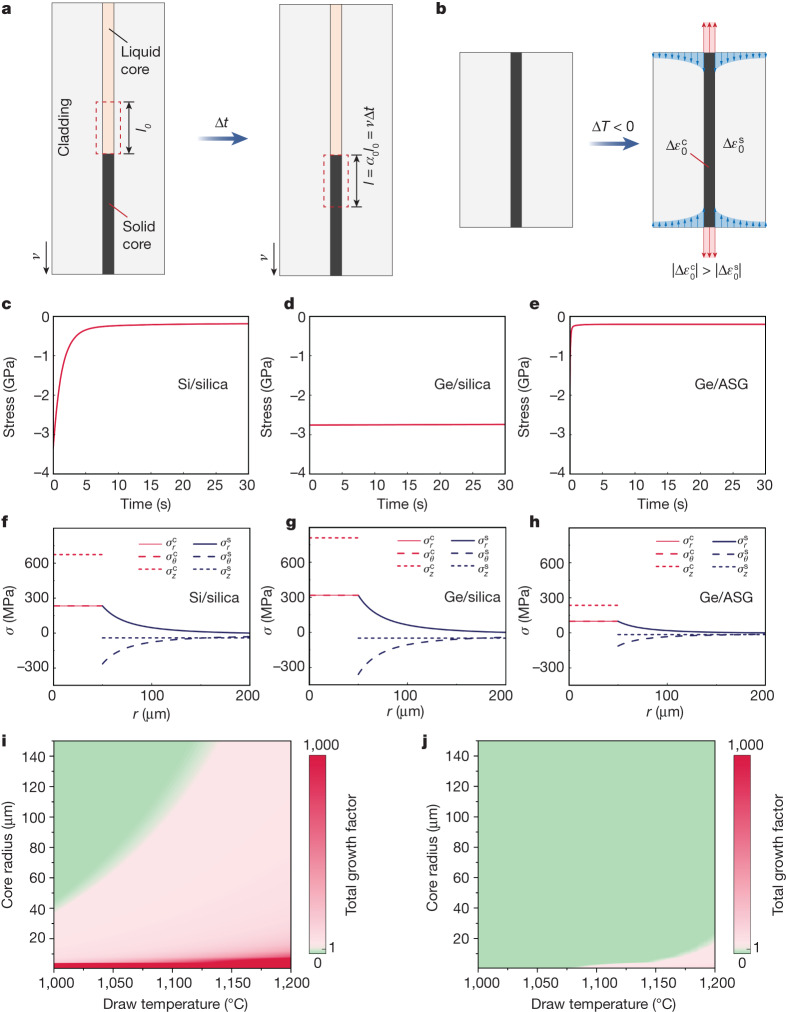
Stress analysis and capillary instability in the molten-core method. **a**, Schematic showing the solidification of a small column of the liquid semiconductor core over a short time of Δ*t*. The position of the liquid–solid interface remains the same in the steady-state thermal drawing process. **b**, Schematic showing the thermal mismatch at the cooling stage. $\Delta {\varepsilon }_{0}^{{\rm{c}}}$ and $\Delta {\varepsilon }_{0}^{{\rm{s}}}$ are the increases in thermal strains of the core and cladding, respectively, under the temperature change Δ*T*. **c**–**e**, Evolution of the maximum principal stress in the solidified semiconductor core of Si/silica (**c**), Ge/silica (**d**) and Ge/ASG (**e**) fibres with a core radius *r*_1_ = 50 μm, fibre radius *r*_2_ = 200 μm and drawing speed *v* *=* 30 mm s^−1^. **f**–**h**, Radial distributions of stress components in the core and cladding of Si/silica (**f**), Ge/silica (**g**) and Ge/ASG (**h**) fibres with *r*_1_ = 50 μm and *r*_2_ = 200 μm after being cooled from the temperature $T=\min \left({T}_{{\rm{m}}}^{{\rm{core}}},{T}_{{\rm{a}}}^{{\rm{clad}}}\right)$ to the ambient temperature (27 °C). The axial stress component of the core (${\sigma }_{r}^{{\rm{c}}}$) has a crucial role in core cracking. **i**,**j**, Contour maps of the total growth factor for Ge/BSG (**i**) and Ge/ASG (**j**) fibres. The green areas indicate the fibre drawing conditions in which the semiconductor core is safe from the growth of capillary instability.

To trace the stress formation in the molten-core method, two stages are identified: the core solidification stage and the subsequent cooling stage (Fig. 2a,b). At the core solidification stage, semiconductor cores are confined by the glass cladding, and the anomalous expansion during the crystallization of Si and Ge cores contributes to the stress formation^30,31^. Then, the core and cladding experience different thermal strains during annealing. Thus, the combination of anomalous expansion and thermal mismatch leads to stresses in semiconductor cores, bringing up the necessity of mechanical design to avoid cracks and fractures, especially when the removal of glass cladding is required.

**Extended Data Fig. 1:**
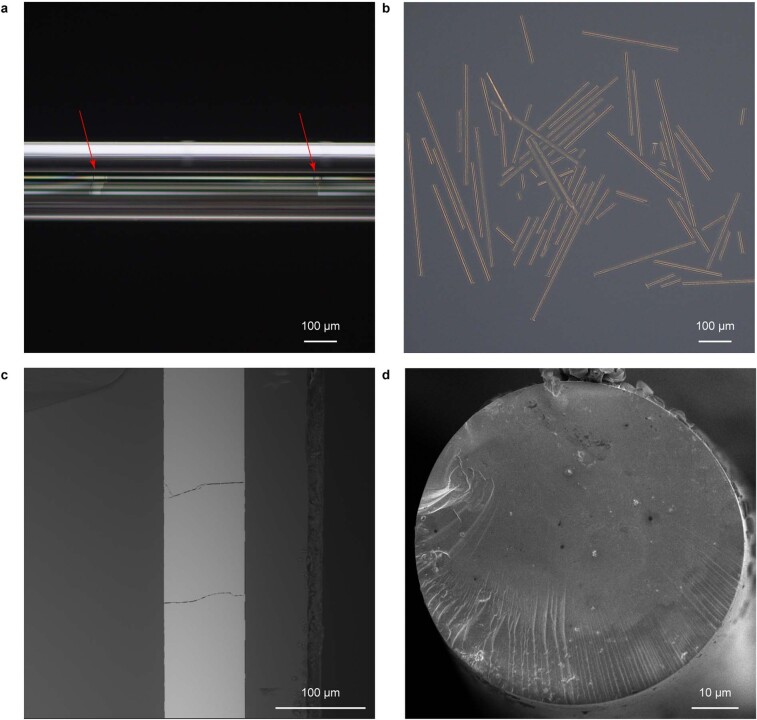
Cracks in the Ge core of Ge/silica fibres. **a**, Cracks observed in as-drawn fibre. **b**, Ge segments after etching away the cladding. **c**–**d**, Lateral and transverse cross-sections show the crack details.

During the core solidification stage, the liquid–solid interface of the semiconductor core remains at the same position after a short period of Δ*t*. The newly solidified portion of the core in Δ*t* with a length of *l* *=* *v*Δ*t*, where *v* is the drawing speed, has experienced a crystallization-induced volume expansion from the corresponding liquid column of the length *l*_0_ before the solidification (Fig. 2a). The volume expansion is equivalent to an axial expansion coefficient *α*_0_ = *l*/*l*_0_ of the core confined within the fibre cladding, which can be determined by finite element simulations. For Ge/silica fibre, cracks were observed in the core^32^, while acceptable core quality was achieved in Si/silica fibre (Extended Data Fig. 1). The different viscoelastic responses of the cladding near the liquid–solid interface during the solidification of the semiconductor core are responsible for these distinct results of Ge/silica and Si/silica fibres. Glass claddings typically exhibit viscoelastic behaviour that relieves stresses at high temperatures, which is closely related to the viscosity. The viscosity of vitreous silica varies widely across more than five orders of magnitude between the melting points of Si (${T}_{{\rm{m}}}^{{\rm{Si}}}=\mathrm{1,410}\,^\circ {\rm{C}}$) and (${T}_{{\rm{m}}}^{{\rm{Ge}}}=938\,^\circ {\rm{C}}$)^33^. As a result, silica glass cladding provides distinct lateral restrictions to the Si and Ge cores. For Si/silica fibre, the internal stresses in the cores are relaxed shortly (<5 s) after the solidification (Fig. 2c), owing to the highly viscous behaviour of the silica glass cladding at the melting point of the Si core. In contrast, stress relaxation in the Ge/silica fibre (Fig. 2d) needs a remarkably longer time (>1,000 s), and high compressive stress remains in the Ge core, owing to the considerable gap between ${T}_{{\rm{m}}}^{{\rm{Ge}}}$ and the annealing point of silica ${T}_{{\rm{a}}}^{{\rm{silica}}}=\mathrm{1,200}\,^\circ {\rm{C}}$. Thus, the persistent high compression brings a substantial risk of forming transverse cracks in the confined Ge core in silica glass cladding (Extended Data Fig. 1). To achieve rational mechanical design, we introduce aluminosilicate glass (ASG) as the cladding material for Ge core fibre. Compared with silica glass, ASG has a much lower annealing point ${T}_{{\rm{a}}}^{{\rm{ASG}}}=795\,^\circ {\rm{C}}$, and exhibits a more viscous behaviour at ${T}_{{\rm{m}}}^{{\rm{Ge}}}$, which contributes to the rapid relaxation (<1 s) of the internal stress in Ge core (Fig. 2e). Selecting ASG as cladding material can save the Ge core from cracking at the solidification stage.

**Extended Data Fig. 2:**
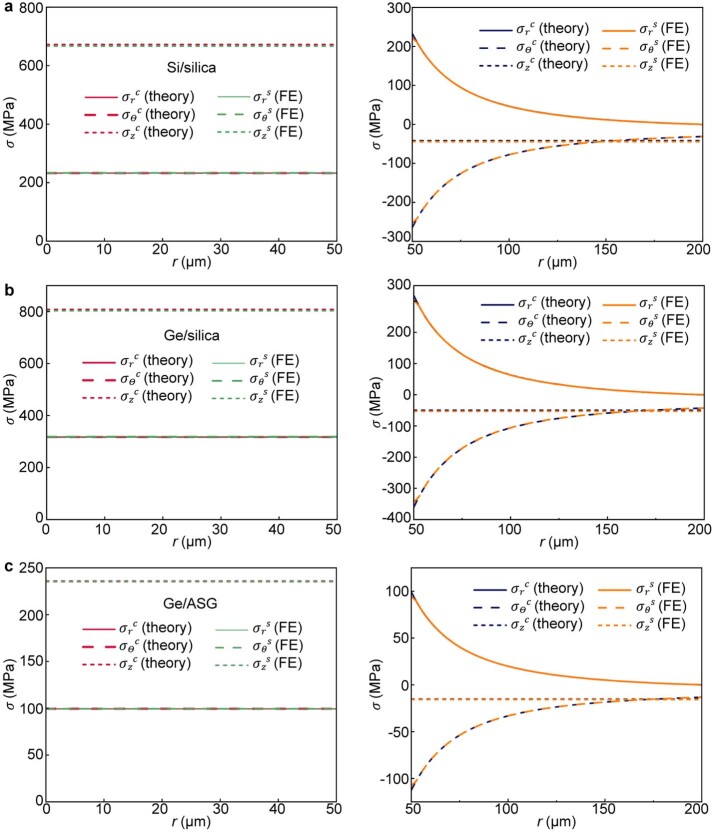
Comparison of theoretical and FE results on the stress distributions. **a**, Si core and silica cladding, **b**, Ge core and silica cladding, **c**, Ge core and ASG cladding after being cooled to the ambient temperature.

When entering the cooling stage, as the crystallized core is drawn away from the furnace, the mismatch in thermal expansion rates of the core and cladding induces additional stresses (Fig. 2b). A mechanical model was developed to study the stress distributions due to such thermal mismatch (Supplementary Note 1). As silica glass has a much smaller coefficient of thermal expansion (CTE) than that of Si and Ge semiconductor cores^34,35^, high axial tensions are generated in the cores after the cooling (Fig. 2f,g). The tensile stresses can promote the propagation of the existing cracks initiated at the solidification stage or even induce new cracks in the Ge core, while the Si core survives, benefiting from its higher strength than Ge. In the Ge/ASG fibre, the cladding has a CTE close to that of Ge (Supplementary Table 1), resulting in small thermal mismatch and axial tension in the core (Fig. 2h). Accordingly, a continuous Ge core without cracks can be obtained. The predicted stress distributions by the model are in excellent agreement with finite element simulation results (Extended Data Fig. 2). In contrast to the Ge fragments from the Ge/silica fibre, a continuous standalone Ge fibre can be obtained after removing the cladding from the Ge/ASG fibre (Supplementary Video 1).

**Extended Data Fig. 3:**
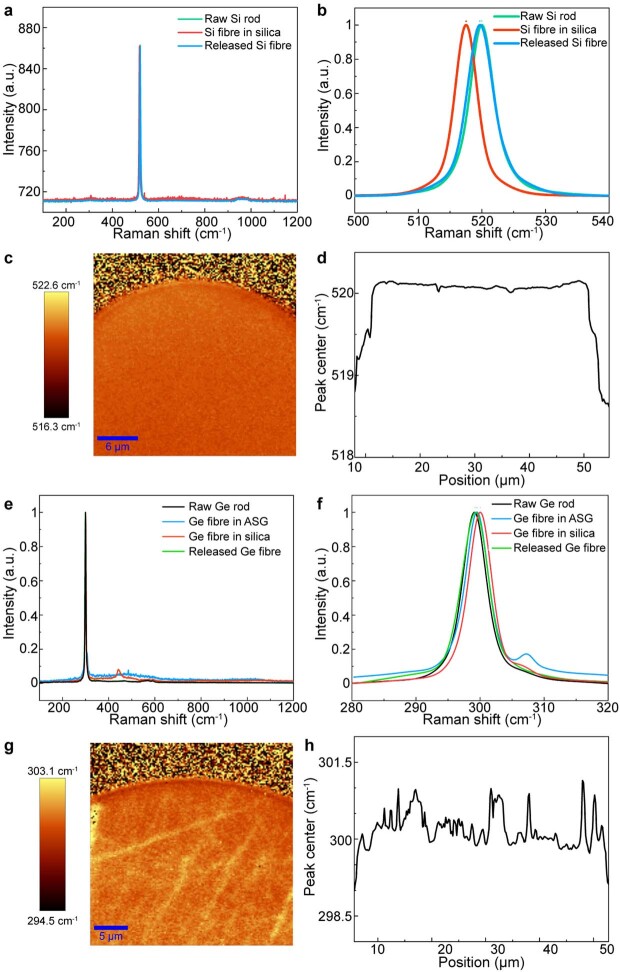
Raman spectra for Si/silica and Ge/silica fibres. **a**, Comparison of Raman spectra between raw Si rod, Si-in-silica, and released Si fibre. **b**, Enlarged image around peak position. Si-in-silica shows a blueshift, indicating the formation of tensile stress, while the released Si fibre has minor residual stress. **c**, Raman mapping of the cross-section. A uniform stress distribution is observed at the inner part, while a ring of tensile stress area around the interface can be observed. **d**, Line scan across the circular cross-section passing through the centre. **e**, Comparison of Raman spectra between raw Ge rod, Ge/silica, Ge/ASG, and released Ge fibre **f**, Enlarged image around peak position. Ge/ASG shows a smaller redshift than Ge/silica, indicating minor compressive stress. **g**, Raman mapping of the cross-section. Owing to the stress formed during cooling, the interface has a smaller redshift than the centre area. Scratches caused by polishing are also observed. **h**, Line scan across the circular cross-section passing through the centre.

The stress analyses indicate that both the volume expansion of semiconductors at the solidification stage and the thermal expansion mismatch at the cooling stage contribute to crack formation in the Ge core confined by silica cladding. The overall stress level in the core can be measured through Raman spectroscopy (Extended Data Fig. 3). Note that the results cannot precisely reveal the pristine stress value due to the necessary polish for sample preparation, but they can serve as a reference for qualitative analysis. The Si and Ge cores in silica cladding have positive and negative residual stresses, respectively (Supplementary Table 2), presumably resulting from the small relaxed compression at the solidification plus an axial tension induced by the thermal mismatch for the Si core, and from the huge compression due to the solidification expansion plus a high axial tension (yet smaller than the compression in magnitude) at the cooling stage for the Ge core. Also note that the as-drawn Ge core in silica cladding was broken into short segments, which might release a part of the accumulated stresses. However, the Ge core in ASG cladding shows a small residual stress because of the lower stress induced at both stages. Our modelling and finite element simulation results provide informative guidance for the fabrication of semiconductor fibres with continuous cores via the molten-core method: to choose the cladding materials with (1) an annealing point close to the melting point of the core and (2) a CTE close to that of the core material.

## Capillary instability

**Extended Data Fig. 4:**
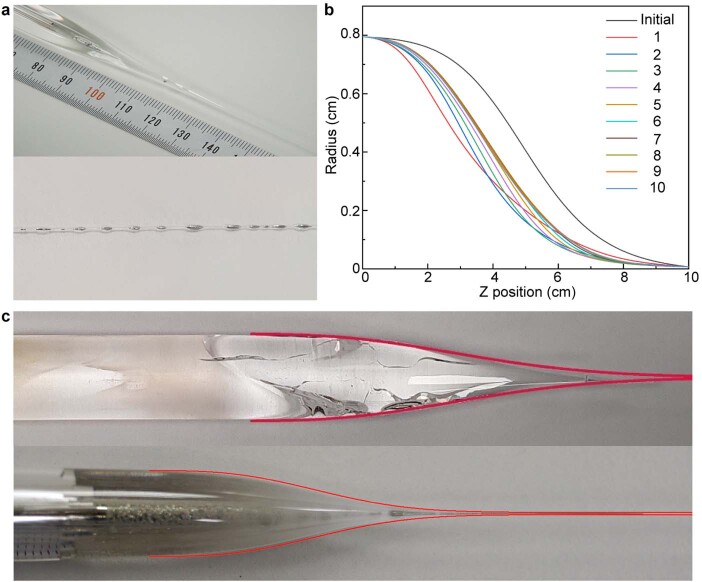
Capillary instability in the molten core method. **a**, Breakup in the core was observed in Ge/BSG system. Ge spheres are observed in the neck-down region as well as perturbated Ge core in the resulting fibre. **b**, Iterative calculation of the neck profile. **c**, Comparison of calculated neck profiles and experimental result. Red curves indicate the calculated neck profiles. Preforms with and without the semiconductor core are quenched to obtain the neck profiles.

Before the solidification of the semiconductor core, the whole fibre remains in the combination of a cylindrical viscous fluid (core) in another viscous fluid (cladding), subject to the capillary instability^36,37^. Capillary instability is unfavoured as it disturbs the continuity and consistency of the cylindrical fibre core at the geometry along both the radial and the axial directions (Fig. 1b and Extended Data Fig. 4a). Thus, a criterion describing the growth of capillary instability related to the draw parameters is essential to study the phenomenon and optimize the process. This can be established by modifying a classical model built for two viscous cylindrical liquids^38,39^ with the consideration of a necking profile (Supplementary Note 2), which can be calculated through an iterative method^40^ (Extended Data Fig. 4b,c). Using this modelling, a total growth factor can be obtained to indicate the magnitude of the exponential growth of capillary instability in the core, thus serving as the criterion. Complete break-up in the core due to capillary instability is expected with a total growth factor far greater than one, while a value much smaller than one indicates stable core geometry^38^.

In addition to silica glass, borosilicate glass (BSG) has also been reported as a cladding material for drawing Ge core fibres^41,42^. The drawing temperature is commonly set to at least 1,000 °C to make sure molten Ge is achieved within the dwell time of travelling in the furnace^41,42^. However, BSG has a low softening point (825 °C) and maintains a comparatively low viscosity at a drawing temperature of 1,000 °C, which brings a high capillary instability growth rate. As a result, perturbations can be observed in the Ge core, especially with slow drawing speeds or small core diameters. To ensure sufficient melting of the Ge core, it is not possible to suppress the growth of perturbations by lowering the drawing temperature. To address this issue, a cladding material with a softening point slightly higher than the melting point of Ge is needed. ASG, with a softening point of 1,005 °C, satisfies this requirement and is a suitable cladding material for hosting a Ge core, considering it also has a suitable annealing point and thermal expansion coefficient to suppress cracking, as discussed above. A comparison of the total growth factor of drawing the Ge core fibre with BSG and ASG is shown in the contour maps (Fig. 2i,j). The results indicate that a broader safe window of drawing temperature and core diameter is enabled by selecting ASG as the cladding material.

## Optoelectronic fibres

**Extended Data Fig. 5:**
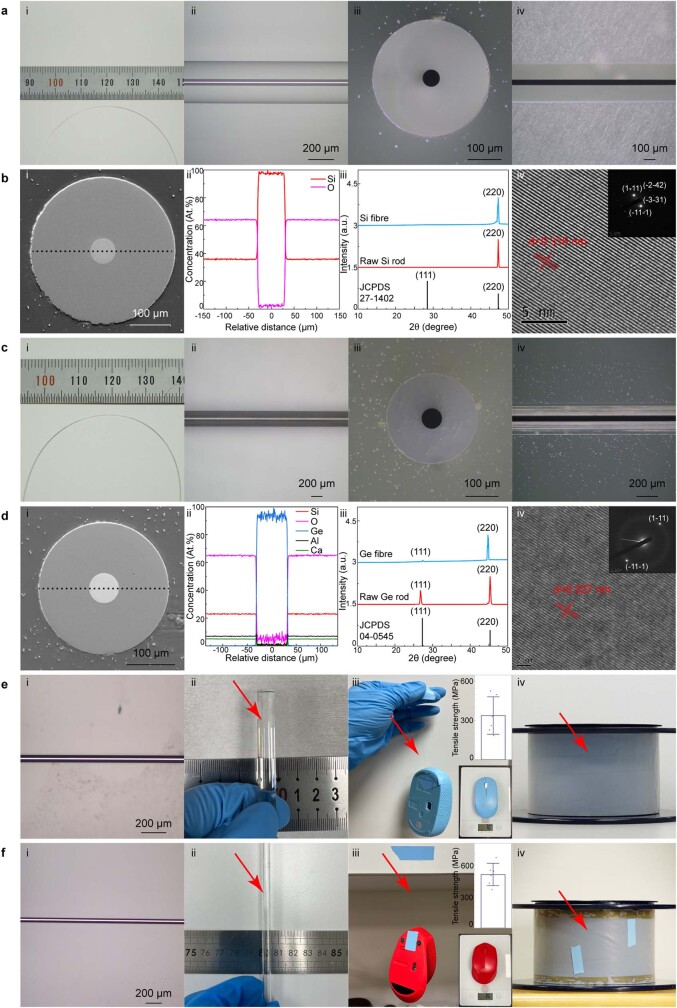
Glass-clad semiconductor fibres and the removal of glass cladding. **a**, Optical images of Si/silica fibres. (i) A bent Si/silica fibre shows flexibility. (ii) No crack/perturbation was observed on the Si core. Dark-field images of the polished (iii) transverse and (iv) lateral cross-sections demonstrate a dense core free of void and crack. **b**, Materials characterization of Si/silica fibres. (i)-(ii) SEM and EDX line scan was applied to the transverse cross-section. (iii) X-ray diffraction patterns confirm the crystallinity of the resulting fibre, and (iv) HRTEM and SAED (inset) images confirm the [Fd$\bar{3}$m1] space group. **c**, Optical images of Ge/ASG fibres. (i) The Ge/ASG fibre under bending. (ii)-(iv) High-quality core free of void and crack was observed. **d**, (i)-(ii) SEM, EDX line scan, (iii) X-ray diffraction, and (iv) HRTEM with SAED pattern in the inset confirm the crystallinity of the resulting fibre with [Fd$\bar{3}$m1] space group. After the removal of glass cladding, standalone semiconductor fibres were achieved. **e**, (i) Standalone Si fibre of 55 μm diameter can be (ii) bent with a bending radius of 4 mm and (iii) easily hold the weight of a mouse (70 g) with the tensile strength of 338.39 ± 143.83 MPa. The mechanical strength allows the (iv) 80 cm-long standalone Si fibre to be safely wrapped on a bobbin. **f**, (i) Standalone Ge fibre of 55 μm diameter can be (ii) bent with a bending radius of 2.5 mm and (iii) easily hold the weight of a mouse (86 g) with the tensile strength of 505.85 ± 105.41 MPa. (iv) The standalone Ge fibre (80 cm) can also be safely collected on a bobbin.

While the glass-clad semiconductor fibres are favoured for optical use^43^, it is challenging to form well defined interfaces between insulators, semiconductors and conductors for in-fibre optoelectronic devices, mainly owing to the limited control on the viscosities of these materials at high processing temperatures^44^. Moreover, the mechanical properties of the glass-clad fibre are compromised, because of the use of glasses. To achieve flexible optoelectronic fibres, the glass cladding was removed by chemical etching, and the standalone Si and Ge fibres were obtained (Extended Data Fig. 5). Next, standalone semiconductor fibre was combined with metal wires, a conducting polymer and an insulating polymer into a single fibre via convergence fibre drawing (see Methods for details). Intimate interfaces between each component were formed at the necking region (Fig. 1e).

**Fig. 3:**
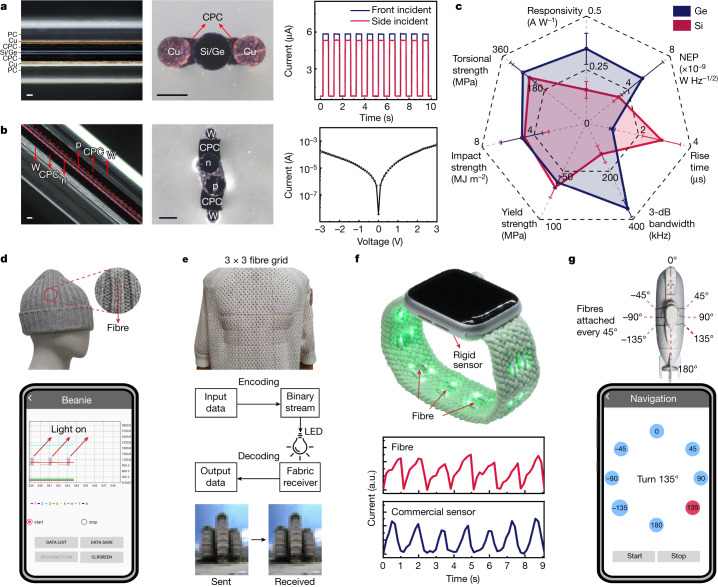
Optoelectronic fibres, fabrics and representative applications. **a**, Left and middle: single-core optoelectronic fibre. Left and middle: optical images of the side-view (left) and cross-section (middle) of the single-core optoelectronic fibre, where the core semiconductor is connected to each copper electrode through a layer of CPC. Right: The resulting optoelectronic fibre shows a pseudo-omnidirectional response that maintains sensitivity for different directions. PC, polycarbonate. Scale bars, 50 μm. **b**, Left and middle: optical images of the side-view (left) and cross-section (middle) of the dual-core p–n junction fibre. Right: the *I*–*V* characteristic of the p–n junction fibre. Scale bars, 50 μm. **c**, Overall performance evaluation of the resulting optoelectronic fibres. The NEP value of the Si optoelectronic fibres is amplified by a factor of ten for visualization. For measurement of responsivity and NEP, *n* = 9 for all cases. For other measurements, *n* = 6 for all cases. All data are presented as mean ± s.d. **d**, Top: the functional beanie used in the demonstration of outdoor use as an assistive wearable device. The interface board was placed inside the beanie tip. Bottom: the signal received by the beanie is visualized in a mobile application. **e**, Top: a functional sweater for indoor Li-Fi communication system. Middle: block diagram of receiving data via the functional sweater. Bottom: a photo of the building (the Learning Hub at Nanyang Technological University) was sent and received via the sweater. **f**, Top: a watchband measures heart pulses via photoplethysmography. Traditionally, a rigid sensor is installed on the backside of the watch. The optoelectronic fibres are woven into the watchband, turning the watchband into a flexible and conformal sensor. Bottom: a comparison of the measured pulse between the fibre and the commercial sensor. **g**, Top: fibre receiver array for underwater visible-light communication system. Fibres were conformally attached to the mini-submarine every 45°, dividing the circumference of the mini-submarine into 8 sections, where each fibre represented a specific angle. Bottom: the command line in the mobile application shows ‘Turn 135°’, when the fibre at 135° receives the signal.

**Extended Data Fig. 6:**
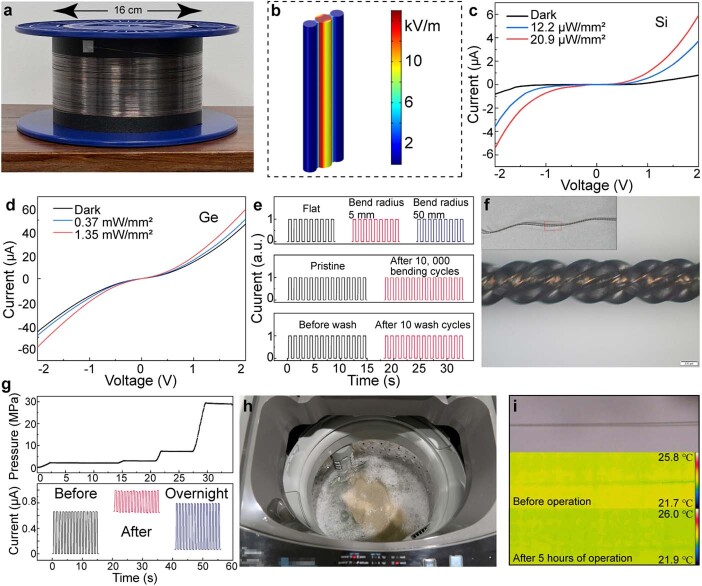
Optoelectronic fibres. **a**, Approximately 50 meters of optoelectronic fibre collected in a single draw. **b**, Simulated electric field distribution, which shows the uniform electric field along the fibre length. **c**–**d**, I-V characteristics of Si and Ge optoelectronic fibres under different illumination conditions. **e**, The photoresponse after bending (bend radius of 5 mm was used in the cyclic testing) and washing tests. **f**, Twisted optoelectronic fibres which still maintain the functionality with the turn density of three turns per mm. **g**, (i) Pressure-time history and (ii) the photoresponse during the compression test. The maximum compressive stress applied was 30 MPa, equal to the pressure underwater at 3,000 meters. The dark current increased while the photoresponse decreased right after the compression. The performance recovered after a night without any additional treatment. **h**, A snapshot of the functional fabric being washed in a washing machine. **i**, After five hours of continuous operation, the temperature of fibre optoelectronic remained unchanged.

The optoelectronic fibre has a rectangular cross-section of 300 µm in width and 200 µm in height with designed back-to-back Schottky contacts in the centre, as shown in Fig. 3a (left and middle) and Extended Data Fig. 6a,b. Figure 3a (right) shows the photoresponse from a Si optoelectronic fibre at front and side incident under 532-nm illumination with a bias voltage of 2 V. The Ge optoelectronic fibre with an identical structure has similar features, but with an extended spectral response due to its smaller bandgap. The current (*I*)–voltage (*V*) characteristics of the Si and Ge optoelectronic fibres are shown in Extended Data Fig. 6c,d. We further studied the variety of in-fibre structures. Figure 3b (left and middle) shows a self-assembled p–n junction fibre. Self-alignment of the two semiconductor fibres was achieved by size reduction of the polycarbonate cladding at the necking region. The *I*–*V* characteristic curve of the p–n junction fibre is shown in Fig. 3b (right). This in-fibre self-assembly technique extends the accessible structures and could increase the utility of fibre electronics.

The Si optoelectronic fibres show a responsivity of 0.16 ± 0.04 A W^−1^ at 532 nm and the value is 0.35 ± 0.07 A W^−1^ at 1,550 nm for the Ge optoelectronic fibres. The level of noise equivalent power (NEP) is 3.12 ± 0.51 × 10^−10^ W Hz^−1/2^ and 5.35 ± 1.97 × 10^−9^ W Hz^−1/2^ for the Si and Ge optoelectronic fibres, respectively. These two types of device respond on the order of microseconds (2.88 ± 0.72 µs and 0.99 ± 0.11 µs for Si and Ge optoelectronic fibres, respectively) and have a 3-dB bandwidth *f*_3dB_ of 129.10 ± 34.89 kHz (Si) and 354.92 ± 34.67 kHz (Ge) (Fig. 3c). A comprehensive performance comparison of the optoelectronic fibres with previously published works and the commercial planar-type photodetector is summarized in Supplementary Table 3.

The optoelectronic fibres are mechanically robust. A stable photoresponse was recorded with a bending radius as small as 5 mm and after repeated bending cycles (Extended Data Fig. 6e). Furthermore, the mechanical strength of the optoelectronic fibres was investigated (Fig. 3c). The tensile strength of the Si optoelectronic fibre (66.80 ± 10.74 MPa) and the Ge optoelectronic fibre (61.59 ± 6.38 MPa) meets the requirement for manual and machine weaving. Although tensile strength is the most important parameter for fibre devices, the impact and torsional strengths are of interest for their practical use in wearable devices. By using one of the most impact-resistant polymers, polycarbonate, as the cladding, the impact strength of the Si and Ge optoelectronic fibres was measured to be 4.74 ± 1.86 MJ m^−2^ and 4.93 ± 1.57 MJ m^−2^, respectively, indicating higher resistance to a break under sudden impact than some conventional fibres (Supplementary Table 3). Moreover, the optoelectronic fibres can withstand a torsional stress of 244.80 ± 70.94 MPa and 272.99 ± 54.16 MPa, respectively, before a break in twisting. Interestingly, the highly twisted optoelectronic fibre with three turns per millimetre still maintained the functionality (Extended Data Fig. 6f). In addition, the recovered photoresponse was recorded with the fibre subject to a compressive stress of 30 MPa (equal to the pressure underwater at 3,000 m) after a night without any treatment (Extended Data Fig. 6g), indicating the potential for underwater applications. Lastly, the washability of the optoelectronic fibre was also demonstrated, and no heat sink was needed during hour-long continuous operation (Extended Data Fig. 6h,i).

## Applications

In addition to operating solely as single-fibre devices, the resulting optoelectronic fibre can be woven into large-scale fabrics, enabling a broad scope of applications (Fig. 3d–g). Here, the Ge optoelectronic fibres are interknitted in a beanie to make it functional (Fig. 3d). An interface board is embedded under the inner top of the beanie. The real-time monitoring of photoresponse on a cell phone is achieved (Supplementary Video 2). In the situation of an alerting sound interrupted by traffic noise or at crossings without the sound-alerting module, the functional beanie acts as an assistive apparel for the visually impaired person. The signals from an infrared light source are received by the beanie and transmitted to a cell phone. Then, the cell phone informs the person whether it is a green or red light through a vibration. The outdoor use of the beanie is demonstrated in Supplementary Video 2. Moreover, the integration of optoelectronic fibres in daily clothing can turn passive apparel into a wearable receiver for a light fidelity (Li-Fi)-based indoor communication system (Fig. 3e). This is demonstrated via the receiving of a photo of a building (the Learning Hub at Nanyang Technological University) using a functional sweater from a modulated light-emitting diode (LED) light source, and a transfer speed of up to 40 KB s^−1^ is achieved (Fig. 3e, bottom). Furthermore, the optoelectronic fibre enables wearable devices for healthcare. A rigid planar-type photodetector, non-conformal to the wrist, is usually installed on the backside of commercial smartwatches to monitor heart rate via photoplethysmography. The optoelectronic fibre is woven into a watchband used as a conformal detector, achieving a similar performance to the commercial photodetectors, in addition to saving the tightly limited inner space of the smartwatch (Fig. 3f). Lastly, the durability under compression makes the waterproof optoelectronic fibre suitable for underwater applications. Eight fibres are conformally attached to a mini-submarine to act as a receiver array in an underwater visible-light communication system (Fig. 3g). Supplementary Video 3 shows the underwater wireless communication between the mini-submarine and cell phone using these fibres.

## Discussion

Stresses in the core materials are mainly induced by the differences in volume changes between the core and the cladding, which originate from the core solidification and mismatched thermal expansion. Perturbation in the core before its solidification is caused by capillary instability. These mechanisms are further supported by modelling and finite element simulation, and such stresses and instability could be relaxed and suppressed upon rational mechanical design through the materials selection and process optimization. The fabrication of high-quality semiconductor fibres suggests that our findings can be used as a guideline to achieve mechanical design for the molten-core method. It could be extended to a broader scope of materials. In addition to materials experiencing anomalous volume expansion similar to that of water–ice change, this guideline is also applicable to materials with solidification shrinkage, which could contribute to the physical separation of the core and the cladding.

Our work has shown that optoelectronic fibres, fabrics and functional apparel can be achieved using the semiconductor fibres, providing a promising pathway to achieve high-performance functional fibres and fabrics^45–47^, as semiconductors are the critical components that primarily govern the devices’ performance. Our findings may bring functional fibres one step further towards unprecedented sensing, actuating, energy converting and computing capabilities^48^.

## Methods

### Materials

Fused silica tubes (purity 99.99%) of 2.1 mm inner diameter (ID) and 12 mm outer diameter (OD) were purchased from Runtu Glass Products. Three sizes (2.1 mm ID and 3.6 mm OD, 9.1 mm ID and 11.3 mm OD, and 13.2 mm ID and 16 mm OD) of ASG tubes (SCHOTT 8253) and rods of 3.75 mm diameter were purchased from SCHOTT AG. Undoped Si (purity >99.999%, resistivity >1.0 Ω cm) and Ge (purity >99.999%) rods of 2 ± 0.127 mm diameter were purchased from Lattice Materials. P- and n-type Si rods (purity >99.999%, resistivity <0.02 Ω cm) of the same size were purchased from the same supplier. Carbon-filled polycarbonate (10^2^−10^6^ Ω sq^−1^) film (thickness 125 μm) was purchased from Boedeker Plastics. Copper (50 μm diameter) and tungsten (30 μm diameter) wires (purity 99.999%) were purchased from Xionglin Metals. Polycarbonate slabs (24 × 8 × 300 mm) were purchased from Tiannuo Polymers. Si and Ge rods were soaked in a 10% hydrofluoric acid solution to remove native oxide before use. Pre-drying of polycarbonate and carbon-filled polycarbonate (CPC) were done in a vacuum oven at 80 °C for 48 h before being used in fibre drawing. Other materials were used without any further treatment.

### Fabrication and characterization of semiconductor fibres

Si rod was inserted into a fused silica tube and sealed in vacuum (1 × 10^−2^ mbar) using an oxyhydrogen flame. Si/silica fibres were fabricated by drawing the preforms at 1,950 °C via the molten-core method, with the feed and draw rates set to 0.002 cm s^−1^ and 3.2 cm s^−1^, respectively. For Ge/ASG fibres, the fabrication started with preform assembling. The ASG tubes of 13.2 mm ID and 16 mm OD were drawn into thinner tubes with 8.9 mm ID and 11.1 mm OD at 1,190 °C. The ASG tubes of 9.1 mm ID and 11.3 mm OD were drawn into three sizes (6.7 mm ID and 8.8 mm OD, 4.9 mm ID and 6.6 mm OD, and 3.7 mm ID and 4.8 mm OD) through the same process. The 3.75-mm-diameter ASG rod was drawn down to 2 mm diameter. The total five sizes of tubes were jacketed to form a preform of 2.1 mm ID and 11.1 mm O, into which a Ge rod was inserted. ASG rods with 2 mm diameter were used in the vacuum (1 × 10^−2^ mbar) sealing of the assembly to finalize the preform. The preform was then drawn at 1,150 °C to obtain Ge/ASG fibres with the feed rate and draw rate set to be 0.002 cm s^−1^ and 3.2 cm s^−1^, respectively. No deoxidizer was used in the process. Standalone Si and Ge fibre were exposed by hydrofluoric acid etching. Before the etching process, glass-clad Si and Ge fibres were cut into segments with a length of 80 cm, limited by the dimension of the acid tank.

Raman spectra were collected using a Witec UHT S300 system (excitation wavelength 532 nm). Fibre lateral- and cross-sections were prepared with embedding samples in epoxy resin (EpoxiCure 2 and EpoThin 2) and then polished with 600, 1200, 2500 and 4000 grit silicon carbide grinding papers. Raman spectroscopy cannot precisely reveal the pristine stress value due to the necessary polish for sample preparation, but it can serve as a reference for qualitative analysis, as the precisely quantitative comparison between modelling (or simulation) and experimental results to determine the individual contribution of solidification expansion and thermal mismatch is currently challenging owing to the extreme working conditions. Through the mechanical optimization for the molten-core method, high-quality glass-clad Si and Ge fibres were obtained. Material characterizations indicate the polycrystalline nature of the fibres with limited oxygen content and free of cracks. Oxygen in the core results from thermally activated dissolution and diffusion from the glass cladding. Extremely low oxygen semiconductor fibres can be achieved by introducing deoxidizer, and laser recrystallization can be applied when the single crystal is desired. Scanning electron microscopy and energy-dispersive X-ray spectroscopy measurements were conducted with the accelerating voltage of 20 kV and working distance of 10 mm using a JEOL JSM-7800F. X-ray diffraction data were extracted by the azimuthal integration of two-dimensional wide-angle X-ray scattering collected by a Xenocs Nanoinxider (sample-detector distance 79.84 mm, wavelength 1.54189 Å, beam size 200 μm, exposure time 60 s and Psi rotation at every 18°). The transmission electron microscopy lamellae were prepared by focused-ion-beam milling. High-resolution transmission electron microscopy and selected area electron diffraction images were collected with a double-tilt holder using a JEOL 2100F 200-kV field-emission transmission electron microscope.

### Fabrication of optoelectronic fibres

Convergence fibre drawing is a modified thermal drawing technique that expands the material selections that are not limited by the process compatibility owing to the drawing temperature. Using this method, the polymers transform into viscous flows and converge to the semiconductor fibres and metal wires that retain solidity when being drawn together down to the fibre dimension.

In our design of an optoelectronic fibre device, standalone Si or Ge fibre was placed in the centre of a transparent polycarbonate cladding and sandwiched by two copper or tungsten wires, while conducting CPC was used to close the gap between semiconductors and metals. Back-to-back Schottky contacts were constructed at the transverse plane between CPC and semiconductors, and further connected with two metal buses to enable decent conductivity both across the microscale transverse plane and along the metre-scale fibre axis. The semiconductor core was slightly thicker than the opaque electrodes to enable pseudo-omnidirectional response to incident light with a beam size larger than the opaque electrodes. It is worth noting that both post-processing techniques, such as laser recrystallization, and semiconductor manufacturing methods, such as doping and lithography, are applicable to the semiconductor fibres and may lead to enhanced performance.

Preparation of the preform used in convergence fibre drawing of the optoelectronic fibres started with the milling of two polycarbonate slabs to create three hemispherical channels with a radius of 2 mm and a distance of 1 mm to each other in the centre, along the preform length. Then, the polycarbonate in the 1 mm space between the three channels was machined off, and two 1 mm square CPC slabs were placed. The preform was then consolidated in a vacuum oven (3 h at 170 °C). The preform was drawn at 300 °C, and the feed rates and draw rates were 0.002 cm s^−1^ and 3.2 cm s^−1^, respectively. Copper wires (for single-core fibre) and tungsten wires (for dual-core fibre) were fed into the two side channels, and Si or Ge fibre (two fibres in the case of dual-core fibre) was fed into the centre channel during the draw.

### Simulations

The finite element analysis for the solidification and cooling stages was implemented with the software suite Abaqus/Standard. In all the simulations, axisymmetric structures were adopted to reduce the computation cost. The parameters used in the simulations are listed in Supplementary Tables 1 and 4.

**Extended Data Fig. 7:**
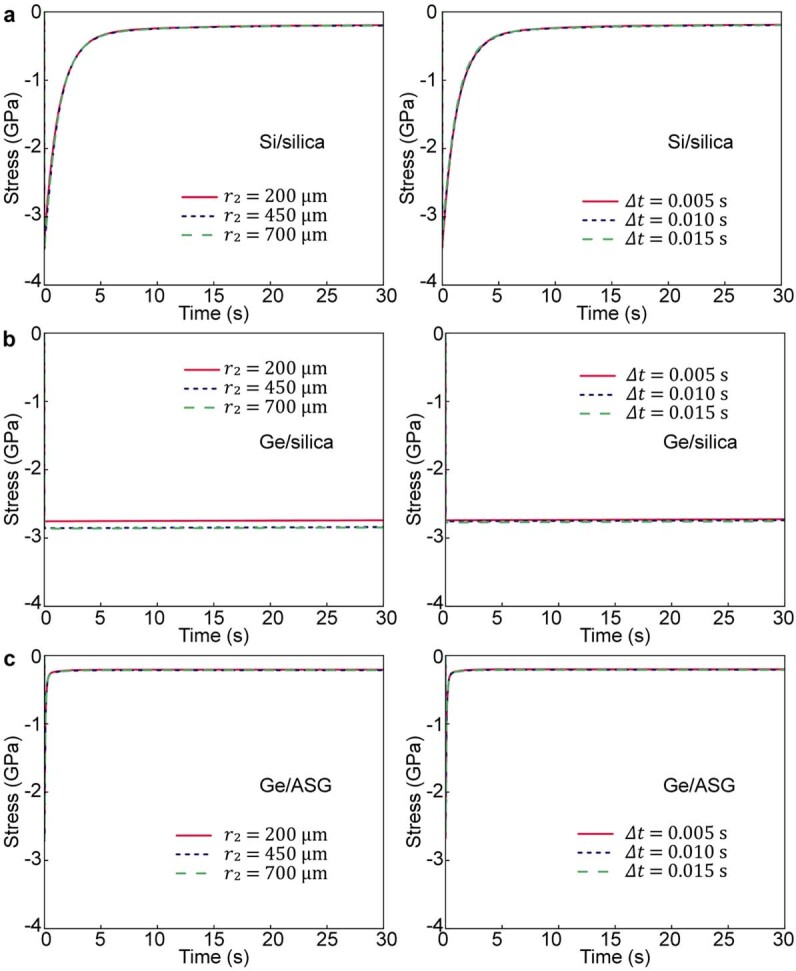
Evolution of the maximum principal stress in the solidified semiconductor core of. (**a**) Si/Silica, (**b**) Ge/Silica, and (**c**) Ge/ASG fibres for different fibre radii (*r*_2_) while keeping the same core radius (*r*_1_ = 50 μm) and for different values of Δ*t*. The FE results show that the choice on the value of Δ*t* has little influence on the stress evolution in the core, if Δ*t* is small compared with the stress relaxation time.

In the first stage, viscoelastic analysis was carried out for the stress distributions after solidification, and the glass claddings were considered as viscoelastic materials with the Maxwell model^33^. The normalized shear relaxation modulus of the claddings was chosen as a value (0.99) close to 1. The volume expansion of semiconductors at solidification was applied in the form of an isotropic eigenstrain induced by a virtual temperature change. As drawing a long fibre at constant velocity is a steady-state process, the liquid–solid interface remained at the same position after Δ*t*. Interfacial sliding was allowed over the portion of the solid core–cladding interface formed during Δ*t*. The choice of the value of Δ*t* does not affect stress evolution in the core if it is small compared with the stress relaxation time (Extended Data Fig. 7). Our conclusions also hold for the other fibres with different ratios of core radius to fibre radius used in the experiment (Extended Data Fig. 7).

In the cooling stage, the viscous effect of the cladding was neglected, considering its much larger viscosities at low temperatures, and accordingly the cladding was modelled as a linear elastic material. Thermal residual stresses started to accumulate in the solidified core when the temperature reached the annealing point of the cladding (${T}_{{\rm{a}}}^{{\rm{clad}}}$), above which the residual stresses would have relaxed rapidly. Therefore, the thermal mismatch was calculated in the temperature range from $T=\min \left({T}_{{\rm{m}}}^{{\rm{core}}},{T}_{{\rm{a}}}^{{\rm{clad}}}\right)$ to the ambient temperature (26 °C). The total thermal strain in this temperature range was obtained by integrating the temperature-dependent data of the linear thermal expansion coefficient for Si, Ge and silicon dioxide^34,35^. Both terminating cross-sections of the fibre were constrained such that the core and the cladding deformed synchronously in the axial direction. The effect of the drawing force is neglected, as it produces much smaller fibre tensions (10–20 MPa) than those induced by the solidification expansion and thermal mismatch.

Capillary instability calculation is described in Supplementary Note 2, and parameters used in the calculations are listed in Supplementary Table 5. Electric field distribution was calculated in the COMSOL electrostatics module with extremely fine mesh.

### Performance characterization

A 532-nm laser diode (Thorlabs DJ-532) and a 1,550,nm InGaAsP laser diode (NEC NX5504EK) were used as the illumination sources. A laser diode current controller (Thorlabs LDC205C), temperature controller (Thorlabs TED200C) and function generator (Agilent 33250 A) were used to operate and modulate the illumination source. The laser beam was focused by a spherical lens and further shaped into an ellipse via a planoconcave lens to cover the whole aperture of the optoelectronic fibres, which was placed in the beam centre. Laser power was monitored by a power meter (Thorlabs PM100). In the measurement of responsivity and *I*–*V* curves, a source meter (Keithley 6517B) was used for power supply and current monitoring. Electrical connections to the fibres were established to the metal wire electrodes exposed by a fibre stripper. A transimpedance amplifier circuit (amplifier OPA380, load resistance 5 kΩ) was used for NEP and rise-time measurements. Waveforms were collected through an oscilloscope (Tektronix DPO5104B), and MATLAB (MathWorks) rise-time and pwelch functions were employed. A bias voltage of 2 V was applied to the fibres in all tests.

Linear translational stages (Thorlabs NRT150) were used in tensile and compression tests with a 20-N force gauge (Yisida DS2-20N) mounted. The impact strength was collected from unnotched Charpy impact tests. Torsional strength testing was conducted on a customized testing machine. The cyclic bending test was performed on a Prtronic FT2000 flexible electronic tester. A commercial washing machine was used in the washability test. Following the ISO 6330 standard, ten washing cycles were applied to the functional fabric.

### Wireless optoelectronic fibre system

A 32 × 48 mm customized printed circuit board (PCB) was used as the interface board for data acquisition, processing and wireless transmission modules. Two GS8554 (four channels) operational amplifiers were used in the transimpedance amplifier circuit, allowing the installation of eight optoelectronic fibres on the PCB. An analogue-to-digital-converter (ADS7828) was used for onboard data processing, and the wireless transmission to cell phone by Bluetooth. A coin cell was used as a power supply. A customized mobile application for visualization was developed.

The functional beanie was achieved by interknitting with eight Ge optoelectronic fibres, which connected to the PCB placed in the inner top space inside the beanie. The demonstration of outdoor use was recorded at a pedestrian crossing at noon on a sunny day with the most intense sunlight of the day using a 30-mW 1,550-nm laser pointer as a signal source at a distance of 1.5 m to the beanie in Supplementary Video 2. Similarly, Si optoelectronic fibres were interknitted into a sweater and used to demonstrate a wearable receiver for an indoor Li-Fi communication system. A photo of the building (the Learning Hub at Nanyang Technological University) was encoded by a customized algorithm into the light from an LED, which was received and converted into electrical signals by the sweater and decoded by a customized algorithm to restore the photo. Mini 532-nm LED strips and Si optoelectronic fibre were integrated together into the watchband. A small portion of the green light related to the volume changes of blood vessels caused by heartbeats was scattered back from the wrist to the optoelectronic fibre and generated an electrical signal reflecting the heart rate. A conventional sensor BIOFY SFH 7070 was used in the same configuration for comparison. In the demonstration of underwater use, Si optoelectronic fibres were conformally (over right-angled steps) glued to the outer surface of the mini-submarine. The PCB interface board was installed beneath the mini-submarine and protected by a waterproof plastic box.

## Online content

Any methods, additional references, Nature Portfolio reporting summaries, source data, extended data, supplementary information, acknowledgements, peer review information; details of author contributions and competing interests; and statements of data and code availability are available at 10.1038/s41586-023-06946-0.

### Supplementary information

Supplementary Information This file contains Supplementary Tables 1–5, Notes 1 and 2, and additional references.

Supplementary Video 1 Comparison of Ge core extracted from Ge/silica fibre and Ge/ASG fibre. The Ge core from the Ge/silica fibre was fractured into short fragments owing to the accumulated stresses. In contrast, an intact Ge fibre can be obtained from the Ge/ASG fibre.

Supplementary Video 2 Demonstration of the functional beanie as assistive apparel in outdoor usage. Optoelectronic fibres were interknitted into a beanie. The real-time communication between the fibres and a cell phone is established by an interface board placed in the inner top space of the beanie. The signals from a 30-mW, 1,550-nm laser pointer at 1.5 m away were recorded by the functional beanie at a pedestrian crossing at noon on a sunny day with the most intense sunlight of the day.

Supplementary Video 3 Underwater usage of optoelectronic fibres. Eight optoelectronic fibres were conformally attached on regular and irregular surfaces with right-angled steps of a mini-submarine, dividing the circumference of the mini-submarine into eight sections, where each fibre represented a specific angle. The interface board was sealed in a waterproof plastic box attached to the bottom. When the fibre representing 135° receives signal from incident light, the command ‘Turn 135°’ is generated and delivered to the mini-submarine.

## Data Availability

All of the data for the main figures and Extended Data figures are available at the DR-NTU (10.21979/N9/BTLRFM).

## Code availability

Custom code used in the fibre-based Li-Fi communication system is available at the DR-NTU (10.21979/N9/BTLRFM).
