# Supplementary material for: High-quality semiconductor fibres via mechanical design
Source: Nature. 2024 Jan 31;626(7997):72–8. doi: 10.1038/s41586-023-06946-0 (PMC10830409; doi:10.1038/s41586-023-06946-0)
Supplement: Supplementary file 1 — This file contains Supplementary Tables 1–5, Notes 1 and 2, and additional references. [file 41586_2023_6946_MOESM1_ESM.pdf]

nature portfolio

https://doi.org/10.1038/s41586-023-06946-0

Supplementary information

High-quality semiconductor fibres via mechanical design

In the format provided by the authors and unedited

Supplementary Information

Supplementary Tables

Supplementary Table 1. Material properties of the aluminosilicate glass (from the technical manual provided by the supplier, the Schott company).

Parameter	Value
Elastic modulus E (GPa)	83
Poisson's ratio v	0.23
Thermal strain (cooling stage) ε_0	-0.359% (with Ge)
Relaxation time τ (s)	0.022 (with Ge)
Normalized shear relaxation modulus g	0.99
Glass transition temperature (˚C)	790
Mean CTE α (10^{-6} K $^{-1}$)	4.7
Viscosity η (Pa·s)	$10^{14.5}$ (750 ˚C)<br>10^{13} (795 ˚C, annealing point)<br>$10^{7.6}$ (1005 ˚C)<br>10^{4} (1305 ˚C)

Supplementary Table 2. Stresses calculated from Raman spectra.

Sample	Raman peak position (cm^{-1})	Stress (MPa)
Raw Si rod	519.97 ± 0.11	0
Si-in-silica	517.51 ± 0.23	470
Released Si	519.71 ± 0.12	50
Raw Ge rod	299.16 ± 0.06	0
Ge-in-silica	300.08 ± 0.14	-235.9
Ge-in-ASG	299.52 ± 0.22	-92.3
Released Ge	299.33 ± 0.14	-43.6

Supplementary Table 3. Comparison of performances of optoelectronic fibres in this work with previously reported optoelectronic fibres, commercial planar device, and conventional polymer and glass fibre material.

Reference	This work	This work	Nat. Nanotechnol.[49]	Nature[50]	Adv. Mater.[51]	Commercial device*	Nylon 6,6[52-55]	Silica fibre[56-58]
**Form factor**	Fibre	Fibre	Fibre	Fibre	Fibre	Planar	Conventional	Conventional
**Material**	Si	Ge	Se	As_2Se_3	Ge	Si	fibre	fibre
**R (A/W)**	0.155	0.347	0.0015	-	0.000038	0.62	N/A	N/A
**Light source (nm)**	532	1550	532	-	1550	960	N/A	N/A
**Rise time (μs)**	2.88	0.99	-	-	1	0.15	N/A	N/A
**NEP (nW/Hz$^{1/2}$)**	3.12	5.35	-	-	-	0.000015	N/A	N/A
**V_{bias} (V)**	2	2	10	50	3	5	N/A	N/A
**Yield strength (MPa)**	66.80	61.59	-	-	-	-	75	5880
**Torsional strength (MPa)**	244.80	272.99	-	-	-	-	88.74	3380
**Impact strength (MJ/m^2)**	4.74	4.93	-	-	-	-	0.16	0.0015

*Thorlabs FDS10X10

Supplementary Table 4. Parameters used in finite element simulations and the mechanical model for stress mismatch analysis.

Parameter	Si	Ge	Silica
Elastic modulus E (GPa)	150	120	70
Poisson's ratio v	0.27	0.27	0.17
Thermal strain (cooling stage) ε_0	-0.471% (with silica)	-0.635% (with silica) -0.525% (with ASG)	-0.055% (with Si) -0.044% (with Ge)
Relaxation time τ (s)			0.48 (with Si) 1767 (with Ge)
Normalized shear relaxation modulus g			0.99
Volumeric solidification expansion	9%	8.3%	
Melting point (˚C)	1410	938	
Annealing point (˚C)			1200[59]

Supplementary Table 5. Parameters used in calculation of total growth factor.

Parameter	Pure silica	Borosilicate glass	Aluminosilicate glass
ε	0.6/0.1	0.6/0.1	0.6/0.1
n	1.47	1.473	1.547
α (cm^{-1})	0.2	0.2	0.2
T_{soften} (˚C)	1600	825	1005
ρ ($g \cdot cm^{-3}$)	2.2	2.23	2.67
γ ($N \cdot cm^{-1}$)	0.003	0.00309	0.004
h ($W \cdot cm^{-2} \cdot K^{-1}$)	0.015/0.03	0.015/0.03	0.015/0.03
K_c ($W \cdot cm^{-1} \cdot K^{-1}$)	0.05	0.05	0.05
C_p ($W \cdot s \cdot g^{-1} \cdot K^{-1}$)	1.0467	1.3	1.3

Supplementary Notes

Supplementary Note 1. Mechanical model for thermal mismatch in the fibre with a single core

An axisymmetric model was developed to consider the problem of thermal mismatch in a fibre with core and cladding structures. The core radius and the fibre radius (i.e., the outer radius of the cladding) are denoted as r_1 and r_2, respectively. The core and cladding materials are linear elastic with Young's moduli E^{c}, E^{s} and Poisson's ratios ν^{c}, ν^{s} where the superscript "c" represents the core and "s" the cladding.

When the core is in liquid phase or the temperature is above the annealing point of the cladding $T_{\mathrm{a}}^{\mathrm{clad}}$, the stress induced by thermal mismatch can be rapidly relaxed and will not be accumulated in the subsequent drawing process. Therefore, the initial stress-free state is chosen as that at the temperature $T = \min\left(T_{\mathrm{m}}^{\mathrm{core}}, T_{\mathrm{a}}^{\mathrm{clad}}\right)$ where $T_{\mathrm{m}}^{\mathrm{core}}$ is the melting point of the core. The total thermal strains $\varepsilon_0^{\mathrm{c}}$ and $\varepsilon_0^{\mathrm{s}}$ are evaluated from the initial temperature down to the ambient temperature. In the cooling stage, no relative displacement is allowed on the interface between the core and cladding, and the cross-section of the fibre in the initial state is assumed to remain flat after thermal deformation. The core and cladding have a common strain ε_z in the axial direction, which, by neglecting the effect of drawing force, can be written as

$$\varepsilon_z = \varepsilon_0^{\mathrm{c}} + \varepsilon_{\mathrm{e}}^{\mathrm{c}} = \varepsilon_0^{\mathrm{s}} + \varepsilon_{\mathrm{e}}^{\mathrm{s}}, \tag{1.1}$$

where ε_{e} denotes the elastic strain in the axial direction. The net axial force on a fibre cross section should vanish, which yields

$$E^{\mathrm{c}}\varepsilon_{\mathrm{e}}^{\mathrm{c}}A^{\mathrm{c}} + E^{\mathrm{s}}\varepsilon_{\mathrm{e}}^{\mathrm{s}}A^{\mathrm{s}} = 0, \tag{1.2}$$

where the cross-section areas of the core $A^{\mathrm{c}} = \pi r_1^2$ and the cladding $A^{\mathrm{s}} = \pi(r_2^2 - r_1^2)$. From Eqs. (1.1) and (1.2), the axial strain of the fibre can be obtained as

$$\varepsilon_z = \frac{E^{\mathrm{c}}\varepsilon_0^{\mathrm{c}}A^{\mathrm{c}} + E^{\mathrm{s}}\varepsilon_0^{\mathrm{s}}A^{\mathrm{s}}}{E^{\mathrm{c}}A^{\mathrm{c}} + E^{\mathrm{s}}A^{\mathrm{s}}}. \tag{1.3}$$

The normal strain components in the radial and tangential directions, denoted as ε_r and ε_θ, respectively, can be divided into the thermal part and elastic part resembling Eq. (1.1). Considering the relation $\varepsilon_r^{\mathrm{c}} = \varepsilon_\theta^{\mathrm{c}}$ in this axisymmetric problem, the radial stress in the core can therefore be obtained from the elastic constitutive equations as

$$\sigma_r^{\mathrm{c}} = \frac{E^{\mathrm{c}}}{(1+\nu^{\mathrm{c}})(1-2\nu^{\mathrm{c}})}\left[\varepsilon_r^{\mathrm{c}} + \nu^{\mathrm{c}}\varepsilon_z - (1+\nu^{\mathrm{c}})\varepsilon_0^{\mathrm{c}}\right]. \tag{1.4}$$

Since stress and strain are uniformly distributed over the cross section of the core, the normal traction and radial displacement of the core at the core-cladding interface are

$$p_{\mathrm{int}} = \sigma_r^{\mathrm{c}}(r = r_1) = \sigma_r^{\mathrm{c}}, \tag{1.5}$$

$$u_{\mathrm{int}} = r_1\varepsilon_r^{\mathrm{c}} = r_1\left[\frac{(1+\nu^{\mathrm{c}})(1-2\nu^{\mathrm{c}})}{E^{\mathrm{c}}}\sigma_r^{\mathrm{c}} - \nu^{\mathrm{c}}\varepsilon_z + (1+\nu^{\mathrm{c}})\varepsilon_0^{\mathrm{c}}\right]. \tag{1.6}$$

The cladding embedding the deformed core can be considered as an elastic tube under a uniform inner pressure p_{int} whose value is to be determined. By adopting the Airy stress function method, the stress fields for such problem can be obtained as

$$\sigma_r^{\mathrm{s}} = \frac{A}{r^2} + B(1 + 2\ln r) + 2C, \tag{1.6a}$$

$$\sigma_\theta^s = -\frac{A}{r^2} + B(3 + 2\ln r) + 2C, \tag{1.6b}$$

where A, B and C are constants. Note that the cladding has a total strain of ε_z in the axial direction as the core does. Therefore, based on Eqs. (1.6), the radial and tangential strain components of the cladding can be calculated as

$$\begin{aligned}\varepsilon_r^s &= \frac{1+\nu^s}{E^s}[(1-\nu^s)\sigma_r^s - \nu^s\sigma_\theta^s] + (1+\nu^s)\varepsilon_0^s - \nu^s\varepsilon_z \\ &= \frac{1+\nu^s}{E^s}\left[\frac{A}{r^2} + (1-4\nu^s)B + 2(1-2\nu^s)B\ln r + 2(1-2\nu^s)C\right] + (1+\nu^s)\varepsilon_0^s - \nu^s\varepsilon_z,\end{aligned} \tag{1.7a}$$

$$\begin{aligned}\varepsilon_\theta^s &= \frac{1+\nu^s}{E^s}[(1-\nu^s)\sigma_\theta^s - \nu^s\sigma_r^s] + (1+\nu^s)\varepsilon_0^s - \nu^s\varepsilon_z \\ &= \frac{1+\nu^s}{E^s}\left[-\frac{A}{r^2} + (3-4\nu^s)B + 2(1-2\nu^s)B\ln r + 2(1-2\nu^s)C\right] + (1+\nu^s)\varepsilon_0^s - \nu^s\varepsilon_z.\end{aligned} \tag{1.7b}$$

From Eq. (1.7a), the radial displacement of the cladding is

$$\begin{aligned}u_r^s &= \int \varepsilon_r^s \mathrm{d}r \\ &= \frac{1+\nu^s}{E^s}\left[-\frac{A}{r} - Br + 2(1-2\nu^s)Br\ln r + 2(1-2\nu^s)Cr\right] + [(1+\nu^s)\varepsilon_0^s - \nu^s\varepsilon_z]r + E,\end{aligned} \tag{1.8}$$

where E is a constant. Since the tangential displacement $u_\theta^s(r,\theta) = 0$, we have

$$\frac{\partial u_\theta^s}{\partial\theta} = r\varepsilon_\theta^s - u_r^s = \frac{4(1+\nu^s)(1-\nu^s)}{E^s}Br - E = 0,$$

which results in $B = E = 0$. Therefore, Eqs. (1.6) and (1.8) become

$$\sigma_r^s = \frac{A}{r^2} + 2C, \tag{1.9a}$$

$$\sigma_\theta^s = -\frac{A}{r^2} + 2C, \tag{1.9b}$$

$$u_r^s = \frac{1+\nu^s}{E^s}\left[-\frac{A}{r} + 2(1-2\nu^s)Cr + E^s\varepsilon_0^s r\right] - \nu^s\varepsilon_z r. \tag{1.10}$$

On the core-cladding interface, the distributions of normal displacement and stress should be continuous, that is,

$$u_r^s(r = r_1) = u_{\text{int}}, \tag{1.11a}$$

$$\sigma_r^s(r = r_1) = p_{\text{int}}. \tag{1.11b}$$

In addition, the outer surface of the cladding is stress-free in the radial direction:

$$\sigma_r^s(r = r_2) = 0. \tag{1.12}$$

Eqs. (1.5), (1.6), (1.9) and (1.10), together with boundary conditions Eqs. (1.11) and (1.12), give closed-form solutions to the distributions of displacements and stresses of both fibre components. From the constitutive equations, the axial stress components of the core and cladding can be obtained as

$$\sigma_z^{\mathrm{x}} = \nu^{\mathrm{x}}(\sigma_r^{\mathrm{x}} + \sigma_\theta^{\mathrm{x}}) + E^{\mathrm{x}}(\varepsilon_z - \varepsilon_0^{\mathrm{x}}), \tag{1.13}$$

where “x” represents “c” or “s”.

Supplementary Note 2. Calculation of capillary instability

Drawing a fibre creates a necking-down region in the heating zone. Peaked at the necking-down region, a convex temperature profile is established along the drawing direction, which softens the amorphous cladding material and melts the core material at a certain length. However, theories on capillary instability of cylindrical threads developed by Rayleigh[60] and Tomotika[61] are not applicable due to the dimension reduction in necking-down region. A neck profile needs to be considered in the modelling. We will first describe how the capillary instability is calculated for the cylindrical case, and then describe how the neck profile is calculated and applied into the calculation of capillary instability in the necking-down scenario.

Rayleigh instability of a viscous fluid fibre[60,61]. Consider an incompressible viscous fluid fibre with a viscosity of η and density of ρ for the case in which the motion is symmetrical about the fibre longitudinal axis z. A perturbation in radius is assumed to be proportional to e^{int}, where t is the time. The perturbation is described spatially along the z axis by e^{ikz} with k as the wavevector. The equations of motion for velocity (u, v, w) in the cylindrical coordinate system are:

$$\frac{\partial u}{\partial t} + u\frac{\partial u}{\partial r} + w\frac{\partial u}{\partial z} = -\frac{1}{\rho}\frac{\partial p}{\partial r} + \frac{\eta}{\rho}\left(\frac{\partial^2 u}{\partial r^2} + \frac{1}{r}\frac{\partial u}{\partial r} - \frac{u}{r^2} + \frac{\partial^2 u}{\partial z^2}\right), \tag{2.1a}$$

$$\frac{\partial w}{\partial t} + u\frac{\partial w}{\partial r} + w\frac{\partial w}{\partial z} = -\frac{1}{\rho}\frac{\partial p}{\partial z} + \frac{\eta}{\rho}\left(\frac{\partial^2 w}{\partial r^2} + \frac{1}{r}\frac{\partial w}{\partial r} - \frac{u}{r^2} + \frac{\partial^2 w}{\partial z^2}\right), \tag{2.1b}$$

where p is the pressure, and the equation of continuity is:

$$\frac{\partial u}{\partial r} + \frac{u}{r} + \frac{\partial w}{\partial z} = 0. \tag{2.2}$$

The Stokes current function ψ is used to describe the flow velocity in a three-dimensional incompressible flow with axisymmetry in fluid dynamics. It is introduced to satisfy the equation of continuity since the fluid is assumed to be incompressible,

$$u = \frac{1}{r}\frac{\partial \psi}{\partial z}, \qquad w = -\frac{1}{r}\frac{\partial \psi}{\partial r}. \tag{2.3}$$

Eliminating p in the equations of motion and introducing the Stokes current function we have:

$$\left(\frac{\partial}{\partial t} + \frac{1}{r}\frac{\partial \psi}{\partial z}\frac{\partial}{\partial r} - \frac{1}{r}\frac{\partial \psi}{\partial r}\frac{\partial}{\partial z} - \frac{2}{r^2}\frac{\partial \psi}{\partial z}\right)\mathrm{D}\psi = \frac{\eta}{\rho}\mathrm{DD}\psi, \tag{2.4}$$

where D denotes the differential operator:

$$\mathrm{D} = \frac{\partial^2}{\partial r^2} - \frac{1}{r}\frac{\partial}{\partial r} + \frac{\partial^2}{\partial z^2}. \tag{2.5}$$

The squares and products of velocity components may be neglected with an assumption of slow motions in Eq. (2.4), and it is reduced to a simpler form:

$$\left(\mathrm{D} - \frac{\rho}{\eta}\frac{\partial}{\partial t}\right)\mathrm{D}\psi = 0, \tag{2.6}$$

The function ψ can be separated into two parts, ψ_1 and ψ_2, since the operators $\mathrm{D} - \frac{\rho}{\eta}\frac{\partial}{\partial t}$ and D are commutative with each other:

$$\mathrm{D}\psi_1 = 0, \tag{2.7a}$$

$$\mathrm{D}\psi_2 - \frac{\rho}{\eta}\frac{\partial \psi_2}{\partial t} = 0. \tag{2.7b}$$

With the assumption of the motions being proportional to e^{int} and e^{ikz}:

$$\psi_1 = \Phi_1 e^{i(nt+kz)}, \tag{2.8a}$$

$$\psi_2 = \Phi_2 e^{i(nt+kz)}. \tag{2.8b}$$

Both Φ_1 and Φ_2 are functions of r only. By combining Eqs. (2.5), (2.7), and (2.8), the equations for Φ_1 and Φ_2 become:

$$\frac{\mathrm{d}^2\Phi_1}{\mathrm{d}r^2} - \frac{1}{r}\frac{\mathrm{d}\Phi_1}{\mathrm{d}r} - k^2\Phi_1 = 0, \tag{2.9a}$$

$$\frac{\mathrm{d}^2\Phi_2}{\mathrm{d}r^2} - \frac{1}{r}\frac{\mathrm{d}\Phi_2}{\mathrm{d}r} - k_1^2\Phi_1 = 0, \tag{2.9b}$$

where

$$k_1^2 = k^2 + \frac{in\rho}{\eta} \tag{2.10}$$

The general solution to Eq. (2.9) is:

$$\Phi_1 = A_1 r I_1(kr) + B_1 r K_1(kr), \tag{2.11a}$$

$$\Phi_2 = A_2 r I_1(k_1 r) + B_2 r K_1(k_1 r), \tag{2.11b}$$

where $I_n(x)$ and $K_n(x)$ are the n-th order modified Bessel functions and A_n, B_n are constants. The general solution to Eq. (2.6) becomes:

$$\psi = \psi_1 + \psi_2$$
$$= \{[A_1 r I_1(kr) + B_1 r K_1(kr)] + [A_2 r I_1(k_1 r) + B_2 r K_1(k_1 r)]\}e^{i(nt+kz)}. \tag{2.12}$$

Rayleigh instability of a viscous fluid fibre in another viscous fluid[61]. The core fluid has a viscosity of η_{core}, density of ρ_{core}, and radius of r_1, while the infinitely thick cladding has a viscosity of η_{clad} and density of ρ_{clad}. The wavelength λ of varicosity (the growth of instability) is in the relationship with k by $\lambda = 2\pi/k$. For the core fluid, since $K_1(0) \to \infty$ we have:

$$\psi_{\mathrm{core}} = \psi_{\mathrm{core1}} + \psi_{\mathrm{core2}} = [A_1 r I_1(kr) + A_2 r I_1(k_{\mathrm{core1}} r)]e^{i(nt+kz)}, \tag{2.13}$$

where

$$k_{\text{core1}}^2 = k^2 + \frac{in\rho_{\text{core}}}{\eta_{\text{core}}}, \tag{2.14}$$

and for the cladding, since $I_1(\infty) \to \infty$ we have:

$$\psi_{\text{clad}} = \psi_{\text{clad1}} + \psi_{\text{clad2}} = [B_1 r K_1(kr) + B_2 r K_1(k_1 r)]e^{i(nt+kz)}, \tag{2.15}$$

where k_1 is given by Eq. (2.10).

In Eqs. (2.13) and (2.15), the constants A_1, A_2, B_1 and B_2 are determined by boundary conditions. Three boundary conditions at the interface of the two fluids are:

1. There is no slipping between the two fluids at the interface. At the interface ($r = r_1$) the velocity components are continuous:
$$u_{\text{core}} = u_{\text{clad}}, \quad w_{\text{core}} = w_{\text{clad}}. \tag{2.16}$$
2. The shear stress is continuous at the interface ($r = r_1$):
$$\eta_{\text{core}}\left(\frac{\partial^2\psi_{\text{core}}}{\partial r^2} - \frac{1}{r}\frac{\partial\psi_{\text{core}}}{\partial r} - \frac{\partial^2\psi_{\text{core}}}{\partial z^2}\right)_{r=r_1} = \eta_{\text{clad}}\left(\frac{\partial^2\psi_{\text{clad}}}{\partial r^2} - \frac{1}{r}\frac{\partial\psi_{\text{clad}}}{\partial r} - \frac{\partial^2\psi_{\text{clad}}}{\partial z^2}\right)_{r=r_1}. \tag{2.17}$$
3. Interfacial surface tension contributes to the difference in the normal stress at the interface ($r = r_1$):
$$\tilde{p}_{\text{clad}} - \tilde{p}_{\text{core}} = \frac{\gamma k (\psi_{\text{core}})_{r=r_1}(k^2 r_1^2 - 1)}{n r_1^3}, \tag{2.18}$$
where γ is the interfacial tension between the two liquids, and $\tilde{p} = -p + 2\eta\frac{\partial u}{\partial r}$ is the normal stress (of the core/cladding) at the interface.

The boundary conditions together with Eqs. (2.13) and (2.15) result in a system of linear equations in terms of the constants A_1, A_2, B_1 and B_2. Nontrivial solutions require the determinant of coefficient matrix to be zero. By neglecting the inertia effect and considering the limit of $\rho_{\text{core}} \to 0$, $\rho_{\text{clad}} \to 0$, the determinant becomes

$$\begin{vmatrix} I_1(kr_1) & kr_1 I_1'(kr_1) & K_1(kr_1) & kr_1 K_1'(kr_1) \\ I_0(kr_1) & I_0(kr_1) + kr_1 I_1(kr_1) & -K_0(kr_1) & kr_1 K_1(kr_1) - K_0(kr_1) \\ \left(\frac{\eta_{\text{core}}}{\eta_{\text{clad}}}\right) I_1(kr_1) & \left(\frac{\eta_{\text{core}}}{\eta_{\text{clad}}}\right) kr_1 I_0(kr_1) & K_1(kr_1) & -kr_1 K_0(kr_1) \\ G_1 & G_2 & K_1'(kr_1) & G_4 \end{vmatrix} = 0 \tag{2.19}$$

where

$$G_1 = \frac{\eta_{\text{core}}}{\eta_{\text{clad}}} I_1'(kr_1) + \frac{\gamma(k^2r_1^2 - 1)}{2inr_1\eta_{\text{clad}}} \frac{1}{kr_1} I_1(kr_1), \tag{2.20a}$$

$$G_2 = \left[\frac{\eta_{\text{core}}}{\eta_{\text{clad}}} I_1'(kr_1) + kr_1 I_1''(kr_1) - I_0(kr_1)\right] + \frac{\gamma(k^2r_1^2 - 1)}{2inr_1\eta_{\text{clad}}} I_1'(kr_1), \tag{2.20b}$$

$$G_4 = K_1'(kr_1) + kr_1 K_1''(kr_1) + K_0(kr_1). \tag{2.20c}$$

The value of n can be determined as a function of kr_1 by this equation. Neglecting the effects of inertia, expanding the determinant in Eq. (2.19) with respect to the fourth row and solving for in, we obtain:

$$in = \frac{\gamma}{2r_1\eta_{\text{clad}}} \varphi(kr_1). \tag{2.21}$$

Here $\varphi(kr_1)$ is treated as the growth factor of instability and given by:

$$\varphi(kr_1) = (1 - k^2r_1^2)\frac{N(kr_1)}{D(kr_1)} \tag{2.22}$$

where

$$N(kr_1) = I_1(kr_1)\Delta_1 - [kr_1 I_0(kr_1) - I_1(kr_1)]\Delta_2, \tag{2.23a}$$

$$\begin{aligned} D(kr_1) = {} & \frac{\eta_{\text{core}}}{\eta_{\text{clad}}}[kr_1 I_0(kr_1) - I_1(kr_1)]\Delta_1 \\ & - \frac{\eta_{\text{core}}}{\eta_{\text{clad}}}[(k^2r_1^2 + 1)I_1(kr_1) - kr_1 I_0(kr_1)]\Delta_2 \\ & - [kr_1 K_0(kr_1) + K_1(kr_1)]\Delta_3 \\ & - [(k^2r_1^2 + 1)K_1(kr_1) + kr_1 K_0(kr_1)]\Delta_4. \end{aligned} \tag{2.23b}$$

In Eq. (2.23), Δ_n is function of kr_1 in determinantal forms as:

$$\Delta_1 = \begin{vmatrix} kr_1 I_0(kr_1) - I_1(kr_1) & K_1(kr_1) & -kr_1 K_0(kr_1) - K_1(kr_1) \\ I_0(kr_1) + kr_1 I_1(kr_1) & -K_0(kr_1) & kr_1 K_1(kr_1) - K_0(kr_1) \\ (\eta_{\text{core}}/\eta_{\text{clad}}) kr_1 I_0(kr_1) & K_1(kr_1) & -kr_1 K_0(kr_1) \end{vmatrix}, \tag{2.24a}$$

$$\Delta_2 = \begin{vmatrix} I_1(kr_1) & K_1(kr_1) & -kr_1 K_0(kr_1) - K_1(kr_1) \\ I_0(kr_1) & -K_0(kr_1) & kr_1 K_1(kr_1) - K_0(kr_1) \\ (\eta_{\text{core}}/\eta_{\text{clad}}) I_1(kr_1) & K_1(kr_1) & -kr_1 K_0(kr_1) \end{vmatrix}, \tag{2.24b}$$

$$\Delta_3 = \begin{vmatrix} I_1(kr_1) & kr_1 I_0(kr_1) - I_1(kr_1) & -kr_1 K_0(kr_1) - K_1(kr_1) \\ I_0(kr_1) & -K_0(kr_1) & kr_1 K_1(kr_1) - K_0(kr_1) \\ (\eta_{\text{core}}/\eta_{\text{clad}}) I_1(kr_1) & K_1(kr_1) & -kr_1 K_0(kr_1) \end{vmatrix}, \tag{2.24c}$$

$$\Delta_4 = \begin{vmatrix} I_1(kr_1) & kr_1 I_0(kr_1) - I_1(kr_1) & K_1(kr_1) \\ I_0(kr_1) & I_0(kr_1) + kr_1 I_1(kr_1) & -K_0(kr_1) \\ (\eta_{\text{core}}/\eta_{\text{clad}}) I_1(kr_1) & (\eta_{\text{core}}/\eta_{\text{clad}}) kr_1 I_0(kr_1) & K_1(kr_1) \end{vmatrix}, \tag{2.24d}$$

The instability growth factor $\varphi(kr_1)$ can be determined using Eq. (2.22).

The wavelength of corresponding instability growth is $\lambda = 2\pi/k$. The instability is suppressed when λ is less than $2\pi r_1$ (kr_1 larger than 1). Thus, kr_1 in the range of 0 to 1 is studied. The actual breakups are determined by the instability with maximum growth factor, and the final spacing of the resulted spheres is the wavelength corresponding to the maximum instability. Therefore, the breakups occur with the wavelength depending on the viscosity ratio and the core diameter.

Criterion for the capillary breakup instability in thermal drawing. The calculation of instability growth factor for a core-clad fibre system is described above. However, the fibre dimension, viscosity, velocity, and temperature vary around the necking region during the thermal drawing process. A dynamic model needs to be considered to raise a criterion for the capillary breakup instability in the thermal drawing process. Due to cross section change induced by necking down, the maximum instability at each z position is different, and the cumulation of instability contributions over the necking region should be considered[62]. As the fibre stays in a viscous state when dwelling time in the furnace, $\tau = 1/in$ is defined as a characteristic time for instability to grow. For exponential instability growth, the perturbation amplitude ε evolves following $\frac{\mathrm{d}\varepsilon}{\mathrm{d}t} = \varepsilon/\tau$. Integrating the equation with respect to time t, the total growth is $\varepsilon \propto e^{\int \frac{\mathrm{d}t}{\tau(t)}}$. The velocity w is position-dependent in thermal drawing and there is $\mathrm{d}z = w(z)\mathrm{d}t$. The total instability growth factor χ can be obtained by:

$$\chi = \int_0^L \frac{\mathrm{d}z}{w(z)\tau(z)}, \tag{2.23}$$

where $z = 0$ is the upper start of the necking region and $z = L$ is the end of the necking region where the final fibre size is reached[63]. The $\tau(z)$ is the shortest growth time at each axial position of the necking region and is calculated by the inverse of Eq. (2.21). The case of a total instability growth factor much smaller than 1 is considered that no capillary instability develops in the draw.

Neck profile calculation. To calculate the total instability growth factor χ, the neck profile $R(z)$ and the distributions of temperature, viscosity and velocity along the axial position are required. A physical model modified based on the one in ref. [63] is considered where the quantities of interest can be obtained by iterative calculation from the coupled momentum and energy equations for a given set of drawing parameters. The influence of core material on the neck profile is neglected due to the small volume ratio of core material, and we assume the radial component of velocity is negligible in comparison with the axial velocity.

Take the starting and finishing point of neck down region as $z = 0$ and $z = L$, the force balance equation can be expressed when the force from surface tension is balanced with the force normal to the surface:

$$\mathbf{S} \cdot \hat{\boldsymbol{n}} + 2\gamma_0 H \hat{\boldsymbol{n}} = 0, \tag{2.24}$$

where $\hat{\boldsymbol{n}}$ is the unit vector, $\mathbf{S}$ the stress tensor, γ_0 the surface tension and H the mean curvature of the surface at $r = R(z)$, defined by:

$$H = \frac{\frac{1}{R} + (R')^2/R - R''}{2[1 + (R')^2]^{\frac{3}{2}}}, \tag{2.25}$$

where $R' = \frac{\mathrm{d}R}{\mathrm{d}z}$, and $R'' = \frac{\mathrm{d}^2 R}{\mathrm{d}z^2}$. Eq. (2.24) can be written as:

$$S_{zz}n_z + S_{rz}n_r + 2\gamma_0 H n_z = 0, \tag{2.26}$$

where n_r and n_z are the radial and axial components of the unit vector $\hat{\boldsymbol{n}}$, expressed as:

$$n_r = \frac{1}{[1+(R')^2]^{\frac{1}{2}}}, \tag{2.27a}$$

$$n_z = -\frac{R'}{[1+(R')^2]^{\frac{1}{2}}}. \tag{2.27b}$$

As the Reynolds number is small, the force balance equation can be written by neglecting the inertial terms as:

$$\rho g + \frac{\partial S_{zz}}{\partial z} + \frac{1}{r}\frac{\partial (rS_{rz})}{\partial r} = 0, \tag{2.28}$$

where g is the gravitational constant. At the starting and finishing points of neck down region, we have the boundary conditions:

$$w = w_{\text{feed}}, \qquad \text{at } z = 0, \tag{2.29a}$$

$$w = w_{\text{draw}}, \qquad \text{at } z = L. \tag{2.29b}$$

Multiplying Eq. (2.28) by $2\pi r dr$, integrating from 0 to $R(z)$, and assuming the elongational and Newtonian flow model with $S_{zz} = 3\eta\frac{\partial w}{\partial z}$, we have:

$$\rho g R^2 + 3(R^2\eta w')' + 4\gamma_0 RHR' = 0. \tag{2.30}$$

Apply the boundary conditions in Eq. (2.29) to the double integration of Eq. (2.30), we have:

$$w = \left[(w_{\text{draw}} - w_{\text{feed}}) + \frac{\rho g}{3}\int_0^L \frac{1}{\eta R^2}\left(\int_0^z R^2 \text{d}z\right)\text{d}z + \frac{4\gamma_0}{3}\int_0^L \frac{1}{\eta R^2}\left(\int_0^z RR'H\text{d}z\right)\text{d}z\right]\frac{\int_0^z \frac{\text{d}z}{\eta R^2}}{\int_0^L \frac{\text{d}z}{\eta R^2}} - \frac{\rho g}{3}\int_0^z \frac{1}{\eta R^2}\left(\int_0^z R^2\text{d}z\right)\text{d}z - \frac{4\gamma_0}{3}\int_0^z \frac{1}{\eta R^2}\left(\int_0^z R'RH\text{d}z\right)\text{d}z + w_{\text{feed}}. \tag{2.31}$$

The heat flux leaving the furnace will be partially absorbed by the preform in the neck down region. While some of the absorbed energy dissipates to the surroundings, part of it will be conducted through the neck down region. Thus, the nonradiative thermal conductivity K_c and radiative thermal conductivity K_r contribute together to the apparent thermal conductivity K. The radiative thermal conductivity K_r is expressed by:

$$K_r = \frac{16\sigma n_0^2 T^3}{3\alpha}, \tag{2.32}$$

where σ is the Stefan-Boltzmann constant, n_0 the refractive index, and α the absorption coefficient. We neglect the transverse temperature gradient as the diameter of preform is less than 15 mm. The heat flux q_z and heat conduction energy balance in the z direction can be expressed by:

$$q_z = -K_c T' - K_r T', \tag{2.33}$$

$$\frac{d[q_z A(z)]}{A(z)dz} + \rho C_p w T' + \frac{2(\varepsilon\sigma(T^4 - T_0^4) + h(T - T_0))}{R} = \frac{2q_{\text{f-n}}}{R}, \tag{2.34}$$

where $A(z) = \pi R^2(z)$ is the area of cross-section, $T' = \frac{\mathrm{d}T}{\mathrm{d}z}$ the temperature gradient, C_p the specific heat capacity, ε the emissivity, T_0 taken to be half of the highest furnace temperature, and $q_{\text{f-n}}$ the heat flux from furnace surface to preform surface. $q_{\text{f-n}}$ is calculated by the integration of the product of emissivity, radiative flux J and shape factor, over the furnace surface:

$$q_{\text{f-n}} = \frac{2\varepsilon r_{\text{fn}}(r_{\text{fn}} - R)}{\sqrt{1 + R'^2}} \int_{-l}^{L_{\text{fn}}-l} \frac{J(j)[-R'(j - z) + (r_{\text{fn}} - R)]}{[(j - z)^2 + (r_{\text{fn}} - R)^2]^2} \mathrm{d}j, \tag{2.35}$$

where r_{fn} is the radius of furnace chamber, L_{fn} the furnace length, and l the length of the section of furnace above the necking region. Radiative flux J can be calculated by:

$$J(j) = J_0 \exp\left[-C\left(\frac{j - \frac{L_{\text{fn}}}{2} + l}{L_{\text{fn}}}\right)^2\right], \tag{2.36}$$

where the axis j for furnace is parallel to z and originates at the beginning point of the necking region. The constant C is obtained from the temperature profile of the furnace, and J_0 can be calculated by:

$$J_0 = \frac{1}{2}\varepsilon_{\text{fn}}\sigma T_{\text{max}}^4 \tag{2.37}$$

where ε_{fn} is the emissivity of the furnace and T_{max} is the highest furnace temperature.

Apply Eqs. (2.32), (2.35), and (2.36) to Eqs. (2.33) and (2.34), we have:

$$q_z = -K_c T' - \frac{16\sigma n_0^2 T^3}{3\alpha} T', \tag{2.38}$$

$$\frac{2q_z}{R} R' + q_z' + \frac{\rho C_p R_0^2 w_{\text{feed}} T'}{R^2} + \frac{2[\varepsilon\sigma(T^4 - T_0^4) + h(T - T_0)]}{R} = \frac{2q_{\text{f-n}}}{R}, \tag{2.39}$$

where R_0 denotes the radius of the preform. From Eq. (2.38) we can deduce that q_z' is:

$$q_z' = -K_c T'' - \frac{16\sigma n_0^2}{3\alpha}[3T^2(T')^2 + T^3 T'']. \tag{2.40}$$

Then Eq. (2.39) becomes:

$$\frac{2R'}{R}\left(-K_c - \frac{16\sigma n_0^2 T^3}{3\alpha}\right)T' - K_c T'' - \frac{16\sigma n_0^2 T^2}{3\alpha}[3(T')^2 + TT''] + \frac{\rho C_p R_0^2 w_{\text{feed}} T'}{R^2} + \frac{2[\varepsilon\sigma(T^4 - T_0^4) + h(T - T_0)]}{R} - \frac{2q_{\text{f-n}}}{R} = 0. \tag{2.41}$$

We can substitute $T = T_1$ and $\frac{\mathrm{d}T}{\mathrm{d}z} = T_2$ into Eq. (2.41), and obtain a system of first-order equations for numerical implementation:

$$\frac{\mathrm{d}T_1}{\mathrm{d}z} = T_2, \tag{2.42a}$$

$$T_2' = \left\{ \begin{array}{c} \frac{2R'}{R}\left(-K_c - \frac{16\sigma n_0^2 T_1^3}{3\alpha}\right)T_2 - \frac{16\sigma n_0^2 T_1^2 T_2^2}{\alpha} + \\ \frac{\rho C_p R_0^2 w_{\mathrm{feed}} T_2}{R^2} + \frac{2\left[\varepsilon\sigma\left({T_1}^4 - T_0^4\right) + h(T_1 - T_0)\right]}{R} \\ -\frac{2q_{\mathrm{f-n}}}{R} \end{array} \right\} \Bigg/ \left(K_c + \frac{16\sigma n_0^2 T_1^3}{3\alpha}\right), \tag{2.42b}$$

The boundary conditions are:

$$T(z) = T_{\mathrm{soften}}, \qquad \text{at } z = 0, \tag{2.43a}$$

$$T(z) = T_{\mathrm{soften}}, \qquad \text{at } z = L, \tag{2.43b}$$

To calculate the neck profile, we first use a hyperbolic tangent function as the input:

$$R(z) = R_0 + \frac{r_2 - R_0}{\tanh[A(L^B - C)]} + \tanh(AC)\tanh[A(z^B - C)] + \tanh(AC)\frac{r_2 - R_0}{\tanh[A(L^B - C)] - \tanh(-AC)}, \tag{2.44}$$

where A, B and C are constants. The temperature profile $T(z)$ can be calculated from Eq. (2.42). From $T(z)$ we can obtain the viscosity profile $\eta(z)$ using the temperature-viscosity relationship of the cladding material. Eq. (2.31) provides a velocity profile $w(z)$, from which we can obtain a new neck profile through the law of conservation of mass. The new neck profile is again used as the input for next calculation, until convergence is reached. The final neck profile is then used in the calculation of total growth factor in Eq. (2.23). MATLAB bvp5c is used to solve the boundary value problem. Less than ten iterations are usually sufficient, depending on the initial input. To verify the model, we calculated the neck profile of a silica preform, with preform feed and fibre draw speed set as 0.001 and 14.4 cm/s, draw temperature set as 1950 °C (Extended Data Fig. 4b). Other parameters used in the calculation are listed in Supplementary Table 5. For ε and h, two values are used for the preform and fibre region, respectively. The first value is used for the section that has a diameter larger than 0.4 cm, and the second value is used for the section with fibre diameter. In the intermediate section, a polynomial is used to smoothly connect the two values. The comparison of the calculated neck profile and experimental result is shown in Extended Data Fig. 4c. The preform was drawn with the same set of draw parameters and then quenched immediately after lifted out from the furnace to maintain the neck profile. Some cracks formed at quenching, which does not affect the result.

References

49 Yan, W., Richard, I., Kurtuldu, G., James, N.D., Schiavone, G., Squair, J.W., Nguyen-Dang, T., Das Gupta, T., Qu, Y., Cao, J.D., Ignatans, R., Lacour, S.P., Tileli, V., Courtine, G., Loffler, J.F. & Sorin, F. Structured nanoscale metallic glass fibres with extreme aspect ratios. *Nature Nanotechnology* **15**, 875-882 (2020). https://doi.org:10.1038/s41565-020-0747-9

50 Bayindir, M., Sorin, F., Abouraddy, A.F., Viens, J., Hart, S.D., Joannopoulos, J.D. & Fink, Y. Metal-insulator-semiconductor optoelectronic fibres. *Nature* **431**, 826-829 (2004). https://doi.org:10.1038/nature02937

51 Wei, L., Hou, C., Levy, E., Lestoquoy, G., Gumennik, A., Abouraddy, A.F., Joannopoulos, J.D. & Fink, Y. Optoelectronic Fibers via Selective Amplification of In-Fiber Capillary Instabilities. *Advanced Materials* **29** (2017). https://doi.org:10.1002/adma.201603033

52 Thomason, J.L., Ali, J.Z. & Anderson, J. The thermo-mechanical performance of glass-fibre reinforced polyamide 66 during glycol–water hydrolysis conditioning. *Composites Part A: Applied Science and Manufacturing* **41**, 820-826 (2010). https://doi.org:10.1016/j.compositesa.2010.02.006

53 Hine, P.J. & Ward, I.M. Hot compaction of woven nylon 6,6 multifilaments. *Journal of Applied Polymer Science* **101**, 991-997 (2006). https://doi.org:10.1002/app.22771

54 Lipkin, J. & Fuchs, E.A. Localized flow of nylon-6,6 rods under torsional loading. *Experimental Mechanics* **29**, 70-74 (1989). https://doi.org:10.1007/bf02327784

55 Bessell, T.J. & Shortall, J.B. The mechanical properties and fracture behaviour of unidirectionally reinforced Nylon 6. *Journal of Materials Science* **12**, 365-372 (1977). https://doi.org:10.1007/bf00566280

56 Matthewson, M.J. & Camilo, G.M. Torsion mechanical parameters of optical fibers. *Proc. SPIE 4215, Optical Fiber and Fiber Component Mechanical Reliability and Testing* 16-27 (2001). https://doi.org:10.1117/12.424371

57 Proctor, B.A., Whitney, I. & Johnson, J.W. The strength of fused silica. *Proceedings of the Royal Society of London. Series A. Mathematical and Physical Sciences* **297**, 534-557 (1997). https://doi.org:10.1098/rspa.1967.0085

58 Corbett, W., Sales, A. & Walton, J.J. Georgia Institute of Technology. Improving the mechanical properties of slip-cast fused silica by fibrous reinforcement (1966). https://doi.org:10.2172/4576365

59 Ojovan, M.I. & Lee, W.E. Viscosity of network liquids within Doremus approach. *Journal of Applied Physics* **95**, 3803-3810 (2004). https://doi.org:10.1063/1.1647260

60 Rayleigh, L. XVI. On the instability of a cylinder of viscous liquid under capillary force. *The London, Edinburgh, and Dublin Philosophical Magazine and Journal of Science* **34**, 145-154 (1892). https://doi.org:10.1080/14786449208620301

61 Tomotika, S. On the instability of a cylindrical thread of a viscous liquid surrounded by another viscous fluid. *Proceedings of the Royal Society of London. Series A - Mathematical and Physical Sciences* **150**, 322-337 (1997). https://doi.org:10.1098/rspa.1935.0104

62 Deng, D.S., Nave, J.C., Liang, X., Johnson, S.G. & Fink, Y. Exploration of in-fiber nanostructures from capillary instability. *Optics Express* **19**, 16273-16290 (2011). https://doi.org:10.1364/OE.19.016273

63 Paek, U.C. & Runk, R.B. Physical behavior of the neck-down region during furnace drawing of silica fibers. *Journal of Applied Physics* **49**, 4417-4422 (1978). https://doi.org:10.1063/1.325495
